# Cross-species comparison of rodent and human decision-making in the Iowa Gambling Task in select neurological and psychiatric disorders: translational approach to examine age- and sex-specific effects of stress and corticolimbic perturbations

**DOI:** 10.3389/fpsyt.2025.1551477

**Published:** 2025-07-22

**Authors:** Varsha Singh, Manjari Tripathi, Sarat P. Chandra, Rohit Verma, Sushil Kumar Jha, Harvinder Singh Chhabra, Mrinmoy Chakravarty, Shambhovi Mitra, Indupriya B, Ankit Jha, Sakshi Sharma, Jyotsna Pandey, Divyanshi Pandey, Insha Shamshad, Ekta Ahlawat, Titli Saha, Chloé César, Suman Jain

**Affiliations:** ^1^ Department of Humanities and Social Sciences, Indian Institute of Technology Delhi (IIT), New Delhi, India; ^2^ Department of Neurology, All India Institute of Medical Sciences (AIIMS), New Delhi, India; ^3^ Department of Psychiatry, All India Institute of Medical Sciences (AIIMS), New Delhi, India; ^4^ School of Life Sciences, Jawaharlal Nehru University (JNU), New Delhi, India; ^5^ Department of Spine and Rehabilitation, Sri Balaji Action Medical Institute, New Delhi, India; ^6^ Department of Social Sciences and Humanities, Indraprastha Institute of Information Technology Delhi (IIIT), New Delhi, India; ^7^ Centre for Design and New Media, Indraprastha Institute of Information Technology Delhi (IIIT), New Delhi, India; ^8^ Indian Spinal Injuries Centre (ISIC), New Delhi, India; ^9^ University of Queensland – IIT Delhi Academy of Research (UQIDAR), Indian Institute of Technology, New Delhi, India; ^10^ School of Interdisciplinary Research (SIRe), Indian Institute of Technology, New Delhi, India; ^11^ Master’s Program, Sorbonne Université, Département du Master Biologie Intégrative et Physiologie (BIP), Paris, France; ^12^ Department of Physiology, All India Institute of Medical Sciences, New Delhi, India

**Keywords:** Iowa Gambling Task (IGT), decision making, animal cognition, rodent models, risk–reward, stress, central nervous system, clinical disorders

## Abstract

**Introduction:**

Rodent models are widely used to understand brain pathologies and address cognitive deficits experienced by humans diagnosed with clinical disorders. However, stark differences in the nervous system and in the environmental demands of rodents and humans make it difficult to translate insights from rodents to humans. Age and sex further increase vulnerability to disorders via experiences marked by neglect, deprivation, threat, and constraining environments instead of care, nutrition, safety, and enriching environment. These differences impact cognitive processing of rewards, risks, and decision-making. Although rodent models allow for investigations of precise brain regions critical for decision-making, such as the prefrontal cortex (PFC), and enable controlled exposure to stress and disorder trajectories, the prefrontal cortex of rodents and humans differ in size, cytoarchitecture, and anatomical–functional organization. This non-analogous structural–functional mapping of brain regions and cognitive deficits result in rodent models that fail to establish causal links of brain pathophysiology and clinical conditions, and the model remains a poor depiction of cognitive deficits experienced by humans. We argue that the Iowa Gambling Task (IGT) is characterized by molecules to behavior, relies on intact cognitive, affective, and motivational systems of inhibitive control involving cortico-limbic circuitry in both humans and rodents.

**Method:**

We conducted a rodent–human task comparison under stress and disruption in the central nervous system (CNS) to link cognitive deficits in poor decision-making with disruptions in brain architecture. A cross-species comparison, accounting for age and sex, was performed on pooled data from human and rodent IGT studies (N = 892; humans = 722; rodents = 170) to examine organism-, age-, and sex-specific decision-making under three levels of stress—psychological stress, CNS perturbation, and limbic perturbation—that can impair decision-making.

**Results:**

The results from four mixed-factor analyses of variances corrected for multiple group comparison showed that stress, CNS perturbation, and limbic perturbations impaired decision making. The adverse effects of psychological stress and CNS perturbations were unique to human task performance, while the adverse effect of limbic perturbations was age-specific in humans and sex-specific in rodents. Infrequent punishment choice was prominent in humans (women), and the healthy group compared to rodents (males) and the CNS perturbed group.

**Discussion:**

Findings suggest that the task might be useful for producing reliable cross-species comparisons of causal mechanisms underlying cognitive deficits in clinical disorders. Preclinical and clinical studies could use the task to reduce the translational gap in neurobiological and clinical neuroscience in ways that might be useful in improving human health.

## 1 Introduction

Cross-species neuroscience emphasizes the need for experimental tasks that facilitate causal explanations from cell-to-circuit level in animal model systems, enhancing our understanding of human brain, behavior, and cognition (1). Despite the inherent differences in the nervous system and the environmental demands, rodents serve as a model system to understand cognitive deficits in neurological and neuropsychiatric disorders. In rodents and human, the pathophysiological alterations of the central nervous system (CNS: brain and spinal cord) will alter the way in which the organism processes information about the internal changes in the body and the external changes in the environment to make goal-directed adaptive choices. This forms the basis of developing animal models of human disease and disorders. The Iowa Gambling Task (IGT: 2) simulates real-life decision-making of selecting choices under uncertainty and risk to maximize rewards and minimize losses with the help of somatic markers. Somatic markers are body states associated with bioregulatory processes of the past that guide decision-making by signaling the value of stimuli/options and guiding choice toward the option that maximizes long-term rewards (3). The IGT is sensitive to brain pathophysiology; hence, it can enable the useful cross-species comparison of structural and functional mapping between rodents and humans under CNS perturbation. The cortico-limbic circuitry engaged in the task is well defined in somatic marker hypothesis and IGT structure (SMH–IGT), which suggests that the ventromedial prefrontal cortex (vmPFC) is critical for emotions and bodily response that guide IGT decision-making (e.g., see 4, 5). Damage to cortical areas such as vmPFC alters somatic inputs (2), and damage to the subcortical areas of medial temporal regions such as amygdala impacts valence and arousal processing (6, 7), the hippocampus impairs emotion-based declarative memory and learning (8), the basal ganglia disrupts choice selection (9, 10), and the cerebellar damage impacts the temporal integration of reward-related actions (11).

For animal models depicting human disorders, it is essential that it has the same etiology or causal mechanisms that underlie human disease and disorder, it has the same phenotype or behavioral manifestation as that observed in humans, and it produces the same response to clinical treatment as observed in humans (12). The IGT is an ecologically valid measure of decision-making, which means that poor performance in the experimental task correlates with poor decisions in real life such as risky behavior observed in substance abuse relapse (13–16). Although causal relationship between poor decision-making and addiction remains ambiguous (17), the task offers ways to probe theoretical questions about causality in clinical disorders. Healthy individuals learn to adjust their choices from immediate risky rewards to delayed safe rewards aided by the somatic clues linked to vmPFC. Damage to the vmPFC disrupts somatic markers, delaying or decreasing the ability to shift to long-term choices. The somatic marker hypothesis (SMH) posits that task performance relies on cortico-limbic processing for brain–body somatic exchange, making the task a useful tool for studying CNS alterations in the cortico-limbic system and cognitive deficits in neurological damage (18). The IGT decision-making relies on emotion (i.e., feeling of risk, rewards, and punishments), working memory (e.g., maintaining a running total of the immediate rewards resulting from a series of choices), and executive control (e.g., inhibiting, shifting from the decks that were rewarding in the initial trials), emotion reactivity (i.e., arousal evoked by risks, rewards, and punishments), episodic memory (e.g., memory of previous rewards and punishments, memory of one large infrequent loss associated with deck B), and autobiographical memory of emotional significance (19). Contrary to the SMH–IGT assumptions, some studies have observed that IGT decision-making in healthy participants is not based on long- vs. short-term rewards (i.e., intertemporal processing), but it is based on the frequency of rewards–punishments with a preference for infrequent punishments (frequency-based processing) (20, 21). Others have used the SMH–IGT framework to further the theoretical discussions regarding structural–functional mapping, for instance, defining the role of the amygdala in IGT decision-making (6) and the role of the dorsolateral prefrontal cortex (dlPFC) in working memory influencing IGT (22). Although the task is widely used in human studies, there is only one study that has concluded that rodent and human decision-making in the IGT might be comparable (23). However, the IGT and the neural circuitry that support task performance have been separately examined in humans and rodents (24–29).

We argue that the SMH–IGT maps the structure–function relationship of the cortico-limbic system critical for cognitive and emotion processing in the IGT (i.e., prefrontal regions and medial temporal structures) and is suitable for cross-species comparison to examine the effect of CNS perturbation on humans and rodents. An intact, unperturbed CNS reflects a well-integrated somatic exchange of information related to emotions (affective valence, arousal) and memory to assess risks and rewards in rodents and humans. However, the effect of the pathophysiological alteration of the CNS perturbation on rodent and human IGT remains unknown. The only study that compared rodent and human IGT decision-making (23) did not test for CNS perturbations on rodent and human decision-making. We examined if IGT is sensitive to CNS perturbation in rodents and humans where we define CNS perturbation at three levels: We first examine the effect of psychological stress on the IGT decision-making. We defined psychological stress as an adverse state caused by a complex higher-order cognition (e.g., anticipatory thoughts and perceptions related to fear–anxiety, risk, loss) that is different from the psychophysiological stress caused by direct, localized body harm or injury. Psychological stress activates the hypothalamic–pituitary–adrenal (HPA) axis, and because the response involves the peripheral system (30, 31), stress effects on CNS might be considered less localized and distinct from those due to CNS perturbations. Stress impacts emotions, adaptive behavior, and the activity of the limbic circuitry, specifically in the amygdala region, and increases vulnerabilities to disorders (30, 32). Ethical challenges hinder the study of stress (30, 31) on decision-making in human participants except in the form of temporary restrictions on sleep, food intake, isolation, time pressure, and social pressures, which are conditions that elicit stress in humans and rodents. Studies suggest that stress due to food restriction impacts the homeostatic balance and increased stress reactivity (cortisol), prominently impairing decision-making in women (33). On the other hand, stress due to anticipating social evaluation (public speaking), experiencing frustration, and helplessness (presented with unsolvable puzzles) caused somatic interference that adversely affected male IGT decision-making via amygdala-mediated stress hormone such as cortisol reactivity (34–36). We examined the effects of 3 h of food restriction in humans and social isolation in rodents to understand how psychological stress influences IGT decision-making.

At the second level, we examine the effect of direct, localized alteration/damage of the brain and spinal cord occurring due to injury/lesion/surgery observed in neurological disorders and in neuropsychiatric disorder of major depressive disorder (MDD). Decision-making deficit is observed across neurological disorders such as traumatic brain injury, multiple sclerosis, and epilepsy (37–40), indicating that localized brain pathology (injury, disease, seizure) has an adverse effect on the IGT. Damage localized to the frontal lobe (41), particularly to vmPFC (42–44) relative to cerebellar damage (11), leads to severe IGT impairment. Despite the critical role that the PFC plays in the CNS as an information processing system, an intact spinal cord carries out the two-way communication between the CNS and the rest of the body (i.e., peripheral nervous system). Damage to the spinal cord impacts the sensory and motor information processing. The extent of loss is proportionate to the level of injury with the cervical level closer to the brain, resulting in the most severe functional impairment. We focused on spinal cord injury as a neurological disorder to examine the effect of CNS perturbation localized to the spinal cord on decision-making. There is only one other study that explored whether impoverished somatic inputs in spinal cord injury localized to the cervical level would impair decision-making in humans and found no impairment in decision-making compared to the healthy group (45). Although rodents are widely used to understand CNS perturbation and regenerative reorganization, the way the cortex is connected to the spinal cord differs drastically between the rodents–humans. The corticospinal connection impacts sensory and motor systems and has challenges in developing human-specific cognitive outcomes associated with spinal cord injury such as phantom sensory inputs and neuropathic pain (46). The IGT produces response to the rewards and punishment, engaging affect processing system and approach-avoidant motivation (47), potentially useful for understanding the role of sensory motor feedback in rodent models that might be comparable to humans. Next, we included a neurological disorder that is characterized by localized CNS perturbation in the form of brain seizures in the frontal and temporal lobe; the most common form of focal epilepsies are the medial temporal lobe epilepsies (the term “mesial” is used for anatomical precision). These commonly involve the limbic circuitry of the amygdala and the hippocampus. Rodent models of temporal lobe epilepsy are more common because these are most pharmaco-resistant with severe cognitive consequences (48). The IGT deficits are observed in frontal and temporal lobe epilepsy (49), but the epilepsy involving the limbic circuitry (amygdala–hippocampal complex) shows severe IGT impairment (50, 51). Inclusion of epilepsy disorder to examine CNS perturbation will help develop rodent models in focal epileptic seizures to understand how rodent–human differences in drug tolerance in drug-refractory epilepsy impact cognitive, affective, and memory systems involving the limbic circuitry. Lastly, we included major depressive disorder as a clinical group to understand CNS perturbation. In contrast to neurological disorders (direct, localized perturbation caused by injury and seizures), a diagnosis of neuropsychiatric disorders like MDD is based on symptoms and deficits because the disorder does not involve a clear biological etiology. There is no clear pathophysiology at cell synapse or circuit levels (52). However, cross-species research suggests that cortico-limbic circuits (cortical regions such as the medial PFC, orbitofrontal PFC, and limbic regions including amygdala) underlie the MDD symptoms (53–55), making MDD well suited to study the effects of CNS perturbations on IGT decision-making.

At the third level, we rely on the SMH–IGT framework and examine the effect of perturbation to the cortico-limbic circuitry that is critical for IGT decision-making, namely, the vmPFC (emotion) and the medial temporal regions of the amygdala (emotion memory) and the hippocampus (episodic memory) (3). The IGT decision-making relies on the integrity of the vmPFC, as the previous sensory and emotional representations (i.e., how emotion information is represented, is codified, and serves as a somatic marker of choices that indicate “good” or “bad” outcomes associated with the options) guided IGT choices (56). We examined if presence of damage to the cortico-limbic circuitry involving frontal and medial temporal regions (e.g., vmPFC in rodents and frontal and medial temporal regions in humans) as specified in the SMH–IGT (3) impairs IGT decision-making. The frontal regions and the medial temporal region forming the “limbic loop” govern multiple memory systems, the amygdala-dependent emotion memory and hippocampus-dependent episodic memory; both are critical for IGT long-term decision-making. Disruption to vmPFC and the medial temporal structures is detrimental to the IGT decision-making (5, 27; see review of limbic system in 57). Additionally, limbic system (frontal and medial temporal regions) alterations of cognitive processing are associated with vulnerability underlying several neurological and neuropsychiatric disorders (58). Despite the crucial role of the limbic circuitry in the SMH–IGT framework and in neurological and neuropsychiatric disorders, the effect of limbic disruption on IGT decision-making has been tested separately in human and rodent models of neuropsychiatric disorders (e.g., 25, 59). There are no cross-species investigations exploring the effect of limbic disruption (vmPFC and medial temporal structures) on IGT decision-making.

Although both neurological and psychiatric disorders demonstrate poor IGT decision-making, findings from neuropsychiatric studies are inconsistent, and the causal link between CNS and cortico-limbic system disruption is elusive, for instance, in schizophrenia, task deficit is not attributed to failure of long-term decision-making but to frequency processing (60, 61). Obsessive–compulsive disorder shows IGT deficit, but the findings are inconsistent (62–64). It is possible that the effect of neuropsychiatric disorder on IGT deficit is heterogeneous because it is sex specific. Although men make more long-term decisions in the IGT (27, 65), women diagnosed with schizophrenia show better IGT performance (select safer long-term choices). Those with major depressive disorder (MDD) show IGT deficit, and those with anxiety disorders perform IGT better. Some causal attributions for women’s task deficit are motivational imbalance (i.e., disproportionate sensitivity to either reward or punishment), emotion dysregulation (i.e., inability to regulate and adjust emotion with the changing reward–punishments), reward-related alteration in frontal cortex (i.e., valence-related frontal asymmetry and hyper/hypo-sensitivity to rewards), and heightened amygdala activity (42, 61, 66–69). Interestingly, some anxiety improve IGT performance (70), specifically in men who meet the Generalized Anxiety Disorder criterion (69). Men show poor IGT decision-making in disorders that show real-life risky, impulsive, short-term decisions, like gambling addiction, alcoholism, and drug addiction (15, 59, 71–73). These observations formed the rationale for examining sex differences in the IGT across organisms and CNS perturbation. Additionally, localized perturbation observed in PFC lesions produces IGT impairment, but age-related pathological PFC alteration shows mixed results on IGT (74, 75); non-pathological aging (old age) shows a deficit in IGT in the absence of other cognitive deficits, indicating that advancing age might impair IGT decision-making (76). Furthermore, aging-related IGT deficits are attributed to the limbic system rather than vmPFC (77). Therefore, we examined age as a covariate to understand the effect of stress, CNS perturbation, and limbic perturbation on decision-making.

Understanding how psychological stress (food restriction and social isolation), localized CNS perturbation in clinical disorders (epilepsy, spinal cord injury, MDD), and limbic perturbation impair decision-making could help understand the association between CNS perturbation and IGT deficit across species (27). Although the entire CNS might not participate in cognitive functions, intact functioning of the CNS is necessary for adaptive functions such as decision-making. CNS perturbations might disrupt somatic cues and the afferent–efferent pathways and adversely impact somatic and cognitive information exchange. The aim is to examine the disruption of information processing in CNS perturbation (less to more localized and disrupted: psychological stress, CNS perturbation in clinical disorders, and limbic disruption) and associated deficit in IGT decision-making. Understanding the effect of CNS perturbation in rodent and humans will provide valuable insights into preclinical and clinical studies in translational and health neuroscience. Clinical investigations of disorders that show sex differences might benefit from a cross-species comparison of CNS perturbations and IGT decision-making (78, 79). There might be an organism- (rodent–human), age- (young–old), and sex-specific (male–female) effect of stress on long-term decision-making. Despite decision-making impairment being a recognized deficit in neurocognitive disorders, no specific task is identified to test decision-making impairment (80). The novelty of our approach is that this is the first human–rodent comparison of CNS perturbation effect on IGT as an experimental task, examined for sex- and age-specific task deficits in neurological and neuropsychiatric disorders. We expected that stress and perturbations would be detrimental to long-term decision-making in an organism- and sex-specific manner accounting for age groups.

## 2 Methodology

Sample and material: Data was pooled from eight studies to create groups that performed the task (six studies with human participants and two rodent studies) (*N* = 895; exclusion: humans = 2; rodent = 1): (a) human vs. rodent and (b) healthy vs. clinical group of CNS perturbations. In the human IGT dataset, the healthy groups comprised participants in the control group and conditions. The clinical group comprised three types of participants: those diagnosed with a neurological disorder and with direct perturbation in the CNS (i.e., seizure, injury to the brain, spinal cord) and those diagnosed with MDD involving CNS alteration. This allowed us to examine decision-making impairment in neurological and psychiatric conditions (see **Table 1**). The human IGT dataset description in **Table 2** indicates the following: healthy participants (368) were pooled from five groups; for the sake of reproducibility, we confirm the following details of these participants: the participants were free from disease and disorders and performed the task under normal testing conditions, i.e., not under the physiological challenge of sleep or food restrictions [baseline data of undergraduates from a sleep loss study (57), age-, sex-, and socioeconomic-status-matched healthy controls for spinal injury participants (47), an undergraduate healthy group in a MDD study (108), an undergraduate healthy group volunteering for a study on emotion and visuospatial processing (43), a satiated group of study on hunger and decision-making (113), a group of undergraduates with temporary food restriction volunteering performed task pre-lunch (107), group diagnosed with MDD (41), a group experiencing spinal cord injury (92), a group diagnosed with epilepsy (62), and a group diagnosed with drug-refractory epilepsy and had undergone brain resection surgery (52).

**Table 1:** Descriptive table of variable/groups and sample size.

Variable/groups	Levels	*N*
Organism type	Human	722
	Rodent	170
Age group	Young	640
	Old	252
Sex	Male	566
	Female	326
Psychological stress	Present	466
	Absent	426
CNS perturbation	Healthy	589
	Clinical	303

**Table 2:** Classification of study-wise sample size based on central nervous system (CNS) perturbation in the human and animal IGT datasets.

	Psychological stress	Neuropathological/localized CNS perturbation
Rodent IGT dataset (*n* = 170)		
Healthy (98)	Absent	Absent
Stressed: maternal separation stress (16)	Present	Absent
Non-specific, sham brain lesion (14)	Present	Present
Task-specific, vmPFC brain lesion (14)	Present	Present
Early stress + sham brain lesion (14)	Present	Present
Early stress + task-specific, vmPFC brain lesion (14)	Present	Present
Human IGT dataset (*n* = 722)		
Healthy (368)	Absent	Absent
Psychological stress: food restricted hunger (107)	Present	Absent
Major depressive disorder diagnoses (41)	Present	Present
Spinal cord injury diagnoses (92)	Present	Present
Epilepsy seizures localized diagnosis (62)	Present	Present
Epilepsy seizures localized diagnoses and post-brain resection (52)	Present	Present

For the rodent task data, healthy rats and rats with stress such as social exclusion via maternal separation were in the non-clinical groups (groups not modeling a clinical condition and without any focal neuropathology), and animals with CNS perturbation as brain lesion (vmPFC or sham lesion) served in the clinical groups. We provide sample descriptions for the human and rodent datasets herewith (see **Table 2**). Rodent IGT dataset: Healthy rodents were pooled (98) from two studies where the animals performed IGT [healthy group from an aging study (78) and healthy group from a sex-differences study (20)] and a group of rodents exposed to post-natal, maternal separation stress for 12 days (16), a group that underwent sham lesions (14), a group that underwent lesions in a region that is critical for IGT, the ventromedial prefrontal cortex (vmPFC: 14), a group that underwent early life stress and sham lesion (14), and a group that underwent early life stress and lesion to the ventromedial prefrontal cortex (14). The effects of psychological stress (hunger in humans and social exclusion in rodents) and stress due to CNS perturbation were examined on IGT (psychological + CNS perturbation of lesion, injury, or surgery to the brain or spinal cord). We first addressed the effects of organism (human vs. rodent), sex (male vs. female), and stress (healthy vs stress group), followed by the effect of CNS perturbation of clinical condition (healthy vs. CNS perturbation). Lastly, we examined the effect of limbic disruption (frontal and medial temporal regions) on IGT decision-making.

### 2.1 Rodent IGT datasets

#### 2.1.1 Rodent Iowa Gambling Task

Wistar rats were trained and tested on the rodent Iowa Gambling Task (rIGT). The animals were housed in separate polypropylene cages with *ad libitum* access to food and water, maintained at 25°C with a 10:14-h light–dark cycle. The wooden maze used consisted of a start area, a choice area, and four arms—two advantageous (C & D) and two disadvantageous (A & B)—distinguished by internal visual cues (10 × 10-cm crosses or circles in black or white). Habituation occurred over 5 days, including exposure to the maze, with sugar pellets as rewards and visual cues in different contexts, ensuring familiarity. The rats who were unresponsive to sugar pellets were excluded to confirm responsiveness to sugar pellets as rewards for the decision-making task. Prior to testing, food restriction reduced the body weight to 95%, using hunger to motivate reward processing while water remained available. The testing spanned 5 days, with 20 trials per day, divided into blocks. Each trial began in the start area, where the rat chose an arm based on rewards (sugar pellets) or punishments (chloroquine tablets). The trials lasted 3 min, and choices were determined by the rat’s full-body entry into an arm. A 30-s inter-trial interval was maintained throughout the testing phase. The choices made by the animals were recorded in the same way as the choices made by the humans (intertemporal long-term decision-making and frequency-based decision-making). The two rodent studies were done in a neurophysiology lab equipped for rodent studies and surgery as a part of a joint doctoral thesis, and the two protocols were approved by the Institutional Animal Ethics Committee of the collaborating institutions.

#### 2.1.2 Aging study

A total of 78 Wistar rats performed the rodent version of the Iowa Gambling Task (rIGT) (all male, old = 39, weight >300 g). The animals performed the decision-making task and memory tasks. The study aimed to compare young and old rats under the effect of age-related cognitive decline and was done as a part of the doctoral thesis of coauthor JP. The protocol that was part of the thesis of JP had approval from the Institute Ethics Committee of the collaborating institute and was overseen by SJ and SKJ.

#### 2.1.3 Sex-differences study

A total of 92 Wistar rats performed the rodent Iowa Gambling Task (IGT) (male = 46, female = 46) to assess sex differences in decision-making. Early stress was induced via maternal separation protocol in 72 rats. The presence of a stress hormone (cortisol) verified the stress levels in the social exclusion and healthy control rodents. The rodents in the lesion groups received excitotoxic lesions targeting the ventromedial prefrontal cortex (vmPFC), which is critical for decision-making processes (i.e., tasks-specific lesions). The sham lesion groups (non-task specific lesions) underwent a similar surgical procedure without the injection of neurotoxin, serving as a control to account for the effects of the surgical intervention in the form of lesioning. This allowed testing the effects of region-specific lesion (vmPFC) and stress on decision-making and anxiety test and was done by coauthor SS as a part of her thesis. There were 28 rodents that had a stereotaxic surgery for lesioning of vmPFC as the circuitry specified in the SMH–IGT framework. This was the task-specific region that was considered as limbic perturbation (SS, unpublished thesis). The protocol (see below) had approval from the Institute Ethics Committee of the collaborating hospital. Lesioning was done by SS and was overseen by SJ.

### 2.2 Human IGT datasets

#### 2.2.1 Hunger state study

A total of 227 healthy undergraduate students volunteered for the study (male = 111, female = 116 females, mean age: 20.65 years; two female students were excluded due to missing data). The participants were tested at pre-lunch time and on an empty stomach (hunger group: no meal consumed for 3 h prior to the testing) or post-lunch (satiated group: had a complete meal at lunch time, 2 h before the testing). The participants filled in their demographic details and VAS (Visual Analogous Scale) for assessing hunger and satiety and were administered the decision-making task. Following the previous work of others, we checked for “hunger” by using VAS ratings to rate hunger, satiety, fullness, and desire to eat (81) and found that the participants in the hunger group stated feeling hungrier than the participants in the satiated group (*p* <.05). The exclusion criterion for the hunger study was to exclude participants with eating disorder, substance use, or psychiatric diagnosis. The participants were excluded from the study if they reported an inability to conform to the protocol (3 h of food restriction for the hunger group and testing within 2 h of food consumption for the satiated group). The participants were excluded from analysis if their data was missing for identification of sex and for VAS response to hunger/satiation. Coauthor CC did part of the data collection as a part of a master’s thesis, and it was completed by coauthor IS as a part of her doctoral thesis. The institute’s ethics committee approved the study protocol.

#### 2.2.2 Spinal cord injury study

Data from two studies were used to examine decision-making in spinal injury: 99 participants comprised of spinal injury and age-matched healthy controls served as a sample for one study of decision-making in spinal injury (spinal injury study 1 = 52 participants with 1-year post-injury, injury level C 4–C 7, male = 91, female = 4; mean age: 28. 31 years; healthy controls = 47, males = 43, females = 4, matched for age, education, and socioeconomic class volunteered for the study). The second study was aimed at understanding cognitive health in spinal injury and recruited an additional 40 participants diagnosed with spinal cord injury (male = 27, females = 13; mean age = 40.20 years). Decision-making in 92 participants diagnosed with spinal cord injury was examined. Both of the studies took place in a designated testing room in a tertiary center for spinal injury. The first study was a part of the doctoral thesis of coauthor SM. The data for the second study was collected by research assistant EA under the supervision of HC. In the spinal cord injury group, the participants with brain pathology and psychiatric or other comorbidities were excluded. Patient Health Questionnaire (PHQ9) was used to assess the mental health of the patients. The participants with cervical damage were included if they had the ability and comfort to use the wrist or adaptive finger for responding to the IGT task via an external mouse. Doctoral student SM supported the wrist at times when needed. The participants’ demographic information was filled in, and they were given bilingual instructions (English and first language) and were requested to carry out decision-making tasks as per the protocol approved by the center’s ethics committee.

#### 2.2.3 Major depressive disorder study

A total of 149 participants served as a sample size in the study (MDD = 41, male = 24, female = 17, mean age: 20.88 years) to examine decision-making. Those who received a diagnosis of major depressive disorder were recruited through referrals from the department of psychiatry in a tertiary hospital. The exclusion criterion was comorbid psychiatric or neurological illness and physical disability. There were 108 healthy undergraduate students (male = 63, female = 44, age: 18–30) recruited for participation in a study recording brain activity during the Iowa Gambling Task using functional near-infrared spectroscopy. The exclusion criterion for healthy controls was diagnosis received for psychiatric or neurological illness, physical disability, incidence of bereavement, or significant events with negative impact as well as the use of prolonged medication that alters mood/affect. Those receiving a diagnosis of major depressive disorder followed an exclusion criterion of comorbid neurological or other psychiatric conditions, and those with mental disability were excluded from the referrals to ensure ability to follow basic instructions. All participants gave demographic information and affect ratings and performed the Iowa Gambling Task. The study was done as part of the thesis of coauthor AJ. The data for healthy groups was collected from a technology institute and was approved by the institute’s ethics committee. The hospital’s ethics committee approved the data collection for the MDD group.

#### 2.2.4 Epilepsy study

The study recruited 115 participants to examine decision-making. The participants were diagnosed with drug-refractory epilepsy (DRE) involving the medial temporal and extratemporal lobes (male = 73, mean age = 24.1 years; female = 42, mean age = 24.5 years; pre-surgery = 62 and post-surgery = 53) and were recruited from the Department of Neurology of a tertiary hospital. Diagnoses were confirmed using video-EEG (vEEG) monitoring and brain imaging (MRI) findings, identifying the patients based on features such as frontal and medial temporal lobe involvement and damage to the hippocampus with and without amygdala damage. Post-surgery participants were tested within 3 months post-operation. Clinical and demographic data were recorded. Affect ratings (mood, disposition) and IGT decision-making tasks were administered individually to assess decision-making; testing time was maintained at prenoon. One female participant’s data was excluded due to the task data being not saved. Coauthors IB and DP collected the data as a part of their doctoral thesis, and the hospital’s ethics committee approved the protocol. For the limbic perturbation, 40 participants with drug-refractory epilepsy who had no clear localized foci as origin of seizure specified in the diagnosis were excluded, six participants who had the occipital or parietal lobe involved in the epileptic seizure and had no involvement of the frontal or medial temporal lobe (no foci, atrophy, anomaly specified for frontal/medial temporal lobe) were excluded, and 68 participants with frontal or medial temporal lobe epilepsy served as a comparison group for limbic loop perturbation on the IGT decision-making (40) (**Table 3**).

**Table 3:** Sample size for rodent–human comparison of task-specific limbic disruption [rodents: ventromedial prefrontal cortex lesion: vmPFC; human: frontal/medial temporal lobe (MTL) epileptic seizures].

Organism type	Sex type	Cortico-limbic disruption	
		Absent	Present
Rodents (ventromedial PFC)	Male (124)	110	14
	Female (46)	32	14
Human (frontal/medial temporal)	Male (442)	395	47
	Female (280)	259	21

### 2.3 Variables and statistical analysis

We analyzed long-term decision-making using the net score method (i.e., cards drawn from safe reward decks C & D minus cards drawn from risky reward decks A & B) for 20 trials, producing five scores for the five blocks of trials (100 trials). It helps to understand how long-term decision-making changes across trials where the rewards and punishments experienced in the initial blocks gradually shift from risky short-term rewards (decks A and B) to long-term rewards (decks C and D). We used a mixed analysis of variance (ANOVA) to examine the main effect of organism, sex, and CNS perturbation group (between-group variable) on the five blocks of net scores that are within-subject variables. We used age as a covariate because we believed that the effects of organism, sex, and CNS perturbation group would differ for young and old age. The first ANOVA addressed if organism (human vs. rodent), sex (male vs. female), and group (healthy vs. stressed: psychological stress + CNS perturbation) served as between-subject variables that impact the net score as within-subject variables (block 1 vs. block 2 vs. block 3 vs. block 4 vs. block 5)—a high net score indicated that more choices were made from the long-term decks compared to the short-term decks. The net scores show a block-wise increase in net scores in the absence of stress. The second ANOVA addressed if organism (human vs. rodent), sex (male vs. female), and group (healthy vs. CNS perturbation) served as between-subject variables that impact the net scores as within-subject variables (block 1 vs. block 2 vs. block 3 vs. block 4 vs. block 5) where CNS perturbation was defined as a clinical group diagnosed with epilepsy, spinal injury, and MDD in humans and had lesions in rodents that impact long-term decision-making. The third analysis addressed if limbic perturbation (vmPFC lesion in rodents and frontal/medial temporal lobe seizure in humans) impacts long-term decision-making. We also examined preference for infrequent punishments (i.e., cards drawn from infrequent punishment decks B & D vs. cards drawn from frequent punishment decks A & C) to examine the “deck B phenomenon” (21) that is prominent in female decision-makers in humans (82) attributed to serotonin-controlled limbic activity in rodents (27).

Multiple group comparisons: We carried out four mixed-factor analyses of variances (ANOVA) to examine the organism and sex-specific effects of healthy group vs. clinical group where we defined the clinical group in three different ways: CNS perturbation, psychological stress + CNS perturbation, and limbic perturbation to test their effect on long-term IGT decision-making. Two additional analyses were conducted to re-analyze the results with data split by the organism to probe organism-specific effects when parity was assumed between rodents and human conditions in the main analyses. Two conditions offered organism-specific follow-up analyses: (a) psychological stress defined in rodents as stress due to social exclusion via the controlled duration of maternal separation and stress in humans defined as 3 h of food restriction producing homeostatic imbalance hunger and (b) disruption of task-specific circuitry was done by comparing limbic perturbation in humans with epileptic seizures of the frontal cortex and medial temporal lobe and limbic perturbation in rodents with lesion to vmPFC. Multiple comparisons of groups on one dependent variable (IGT decision-making) increases the possibility of type I error (false positive), especially in the absence of clear theoretical reasons to guide the analysis. Although we stated the theoretical rationale for testing the groups, we further minimized the risk of type I error by applying a strict Bonferroni correction and interpreted the results by adjusting the alpha level to 0.0125 for determining statistically significant effects of groups on block-wise IGT scores (three groups: healthy vs. CNS perturbation, healthy vs. stress + CNS perturbation, and limbic perturbation present vs. absent compared on block-wise decision-making and the fourth analysis that addressed frequency-based deck choices).

## 3 Results

**Figure 1:**
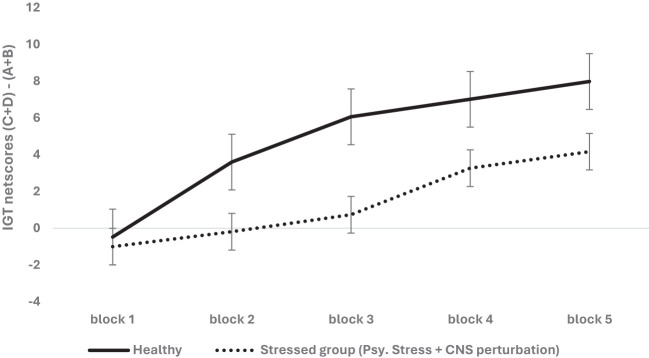
The two-way interaction of group type (healthy vs. stressed group comprised of psychological stress + physiological stress of CNS perturbations) and block-wise IGT net score was significant, demonstrating the adverse effect of psychological stress + psychophysiological stress of CNS perturbations on long-term decision-making (i.e., low IGT net scores). The healthy group chose more cards from the safe decks (C & D) than the risky decks (A & B) compared to the stressed group. Error bars show the standard error.

We first examined the effect of stress on decision-making where stress was defined as psychological stress (hunger in human and social exclusion in rodents) with psychophysiological stress of CNS perturbation in the form of alteration of the CNS confirmed via a neurological or neuropsychiatric diagnosis in humans (e.g., seizure, lesion, injury to the CNS) and lesions in rodents. We analyzed the effect of organism, sex, and stress (psychological stress + psychophysiological stress of CNS perturbations) on long-term decision-making with age as a covariate. We used a mixed analysis of variance with 2 (organism: rodent vs. human) × 2 (sex: male vs. female) × 2 stress condition (healthy vs. stressed) × 5 blocks of IGT (block 1, block 2, block 3, block 4, block 5) with block-wise net scores as the within-subject variable and age as the covariate. The Huynh–Feldt correction was applied, and the results indicated a significant effect of the blocks: *F* (3.42, 3,027.18) = 65.44, *p* = .000, partial *η* ^2^ = .069. The covariate effect was significant, indicating that young and old age groups performed differently: *F* (3.42, 3,027.18) = 4.91, *p* = .001, partial *η* ^2^ = .006 (net scores of young > old). There was no effect of organism or sex type, and no other interactions were significant. Stress significantly impaired long-term decision-making: *F* (3.42, 3,027.18) = 5.98, *p* = .000, partial *η* ^2^ = .007 (healthy: mean 1 = -.47, mean 2 = 3.60, mean 3 = 6.09, mean 4 = 6.82, mean 5 = 7.98; stressed: mean 1 = -1.0, mean 2 = -.19, mean 3 = .73, mean 4 = 3.24, mean 5 = 4.16) (see **Figure 1**). All effects were significant at the adjusted alpha level set for multiple-comparison correction (*p* = .0125).

**Figure 2:**
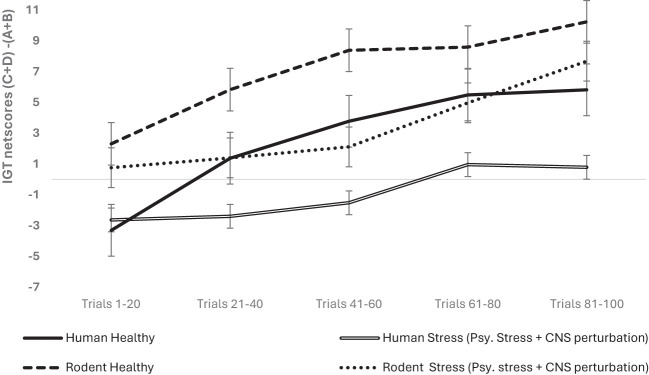
The organism-split analysis of psychological stress + CNS perturbation showed that its effect was significant only in humans, not in rodents. Long-term decision-making improved across blocks for both organism types, but the effect of group with stress + CNS perturbation showed impaired long-term decision-making only in human participants, and psychological stress might have an effect that is unique to humans. The error bars show the standard error.

The organism- and condition-specific differences for the psychological stress condition in humans (hunger) and rodents (isolation) were examined as potentially inequitable. A follow-up analysis was done with the data split for organism type (human vs. rodent), and the effect of sex and stress (i.e., stress + CNS perturbation) on long-term decision-making with age as a covariate was re-examined for the organism-specific effect. We used a mixed analysis of variance with data split on organism type: rodent and human for examining 2 (sex: male vs. female) × 2 stress condition (healthy vs. psychological stress + CNS perturbations) × 5 blocks of IGT (block 1, block 2, block 3, block 4, block 5) with block-wise net scores as the within-subject variable. In humans, the results indicated that long-term decision-making improved across blocks: *F* (3.39, 2,434.50) = 94.49, *p* = .000, partial *η* ^2^ = .116; age impacted long-term decision-making: *F* (3.39, 2,434.50) = 4.71, *p* = .002, partial *η* ^2^ = .007; sex had no significant effect: *F* (3.39, 2,434.50) = 1.05, *p* = .37; and stress impaired long-term decision-making: *F* (3.39, 2,434.50) = 22.05, *p* = .000 partial *η* ^2^ = .03. The interaction of sex and stress was not significant: *F* (3.39, 2,434.50) = 1.07, *p* = .36. In rodents, long-term decision-making improved across blocks: *F* (3.50, 578.99) = 22.36, *p* = .000, partial *η* ^2^ = .119, and the effect of age was not significant: *F* (3.50, 578.99) = 2.28, *p* = .067, partial *η* ^2^ = .014. There was no effect of sex: *F* (3.50, 578.99) = 1.36, *p* = .24; stress (i.e., psychological stress + CNS perturbation) did not impair long-term decision-making in rodents: *F* (3.50, 578.99) = 1.82 *p* = .132; and the interaction of sex and stress was not significant: *F* (3.50, 578.99) = .64, *p* = .60 (see **Figure 2**). The effects survived the correction for multiple comparisons (met the adjusted level of statistical significance). Thus, the analysis of data split by organism type showed that stress (psychological and CNS perturbation) might be detrimental for human IGT decision-making.

**Figure 3:**
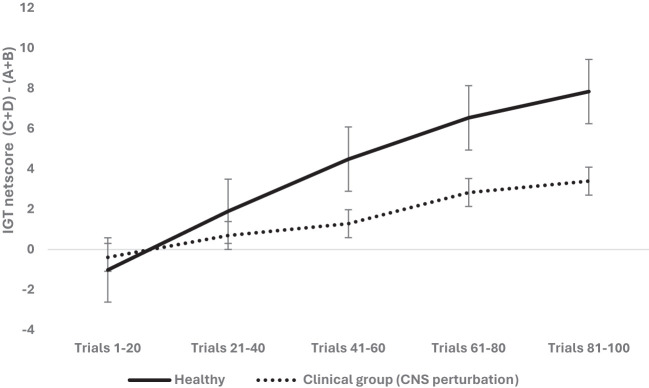
The two-way interaction of group type (healthy vs. clinical group of CNS perturbation) and block-wise IGT net score was significant, demonstrating that CNS perturbation (lesions in rodents and clinical diagnosis of major depressive disorder, spinal injury, epilepsy in humans) was detrimental for long-term decision-making. The healthy participants made more choices from the safe decks (C and D) than from risky decks (A and B) compared to those with CNS perturbations. The error bars show the standard error.

**Figure 4:**
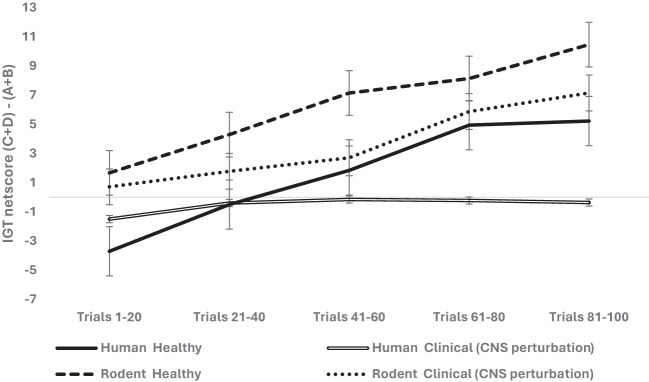
The three-way interaction of organism (rodent vs. human), group (healthy vs. clinical with CNS perturbation), and block-wise IGT net score was significant. The long-term decision-making was higher in healthy rodents compared to healthy humans (the high net score of healthy rodents indicated that more choices were made from safe arms C and D compared to risky arms A and B). The clinical group with CNS perturbation showed low net scores, indicating that humans diagnosed with clinical conditions (spinal injury, epilepsy, major depressive disorder) showed poor long-term decision-making compared to rodents with CNS perturbations (i.e., rodents with lesions made more choices from long-term reward arms C and D compared to risky arms A & B). The error bars show the standard error.

We examined next the effect of CNS perturbation on decision-making and used a mixed analysis of variance with 2 (organism: rodent vs. human) × 2 (sex: male vs. female) × 2 (healthy vs. clinical group with CNS perturbation) × 5 blocks of IGT (block 1, block 2, block 3, block 4, block 5) with block-wise net scores as the within-subject variable (see **Table 2** for groups and levels) and age as the covariate (median-based cutoff on years/weight used to create young and old groups). Mauchly’s test was significant (*χ* ^2^ = 236.04, *p* = .000), and Huynh–Feldt correction was applied based on the epsilon value. The results indicate that the effect of blocks was significant and long term decision-making improved across the five blocks: *F* (3.53, 3,122.49) = 57.216, *p* = .000, partial *η* ^2^ = .061 (mean 1 = -.70, mean 2 = 1.29, mean 3 = 2.88, mean 4 = 4.68, mean 5 = 5.62). The effect of age was not significant: *F* (3.53, 3,122.49) = 1.46, *p* = .21. The effect of organism was insignificant: *F* (3.53, 3,122.49) = 2.83, *p* = .029; it failed to meet the corrected alpha value for statistical significance for multiple comparisons (*p* > 0.0125) (human: mean 1 = - 2.6, mean 2 = -.44, mean 3 = .83, mean 4 = 2.37, mean 5 = 2.43; rodent: mean 1 = 1.19, mean 2 = 3.03, mean 3 = 4.93, mean 4 = 7.01, mean 5 = 8.81). The effect of sex was not significant: *F* (3.53, 3,122.49) = 1.71, *p* = .152. The CNS perturbation significantly impaired long-term decision-making: *F* (3.53, 3,122.49) = 9.61, *p* = .000, partial *η* ^2^ = .011 (healthy: mean 1 = - 1.01, mean 2 = 1.90, mean 3 = 4.49, mean 4 = 6.54, mean 5 = 7.84; clinical: mean 1 = -.38, mean 2 = .69, mean 3 = 1.28, mean 4 = 2.83, mean 5 = 3.39; see **Figure 3**). The interaction of sex and organism was not significant: *F* (3.53, 3,122.49) = 1.27, *p* = .27. The interaction of sex and CNS perturbation was not significant: *F* (3.53, 3,122.49) = 1.10, *p* = .34. However, the interaction of organism and CNS perturbation was significant: *F* (3.53, 3,122.49) = 4.63, *p* = .002 partial, *η* ^2^ = .005, such that human long-term decision-making showed an adverse effect of CNS perturbation (human healthy group: mean 1 = - 3.70, mean 2 = -. 49, mean 3 = 1.83, mean 4 = 4.94, mean 5 = 5.22; human CNS perturbation/clinical group: mean 1 = - 1. 49, mean 2 = -.39, mean 3 = -.15, mean 4 = -. 22, mean 5 = -. 35) compared to rodents’ decision-making under CNS perturbation (rodent healthy group: mean 1 = 1.67, mean 2 = 4.29, mean 3 = 7.15, mean 4 = 8.14, mean 5 = 10.46; rodent clinical group: mean 1 = .71, mean 2 = 1.78, mean 3 = 2.71, mean 4 = 5.88, mean 5 = 7.15) (please see **Figure 4**). The interaction effect of sex, CNS perturbation, and organism on long-term decision-making was insignificant: *F* (3.53, 3,122.49) = 1.18, *p* = .31. A follow-up analysis helped examine organism-specific adverse effects of localized CNS perturbation of lesion observed in rodents and the adverse effect of CNS disruption due to neurological and neuropsychiatric disorders in humans (MDD, spinal injury, epilepsy seizures, epilepsy surgery). The results for humans indicated that improvement in IGT occurred over trials. There was no effect of age and sex, but CNS perturbation showed an adverse effect on decision-making: *F* (3.53, 2,527.85) = 33.91, *p* = .000, partial *η* ^2^ = .045. Rodent decision-making improved with trials. The effect of age, sex, and CNS impairment was not significant (*p* >.05).

**Figure 5:**
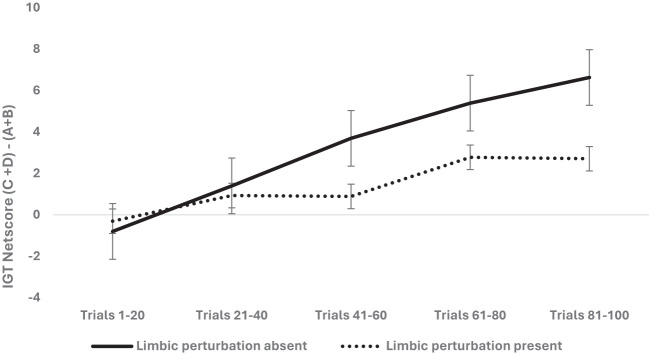
The two-way interaction was significant, indicating that the presence of limbic perturbation [vmPFC lesion (rodents) and frontal/medial temporal seizures (humans)] significantly impaired the long-term decision-making in the IGT, showing low net scores (more choices from risky decks A and B) compared to the group of rodents and humans with absence of limbic perturbation.

The next step was to examine if limbic perturbation (prefrontal and medial temporal structures), specified in SMH–IGT framework and implicated in emotion and memory system, impairs IGT long-term decision-making. We analyzed limbic disruption where the ventromedial prefrontal cortex (vmPFC) lesion in rodents and human participants diagnosed with epilepsy seizures in the frontal or medial temporal lobe were grouped as limbic perturbation. We used a mixed analysis of variance with organism type (rodent vs. human) × 2 (sex: male vs. female) × 2 limbic disruptions (absent vs. present) × 5 blocks of IGT (block 1, block 2, block 3, block 4, block 5) with block-wise net scores as the within-subject variable and age as the covariate. Huynh–Feldt correction was applied, and the results indicated a significant effect of the blocks: *F* (3.44, 3,036.01) = 32.95, *p* = .000, partial *η* ^2^ = .036. The difference between young and old age groups was significant: *F* (3.44, 3,036.01) = 8.22, *p* = .000, partial *η* ^2^ = .009. The effect of organism type was not significant: *F* (3.42, 3,027.18) = 1.76, *p* = .13. The effect of sex was insignificant: *F* (3.42, 3,027.18) = 2.87, *p* = .022, partial *η* ^2^ = .003 (adjusted level of significance 0.0125). The effect of limbic disruption was significant: *F* (3.44, 3,036.01) = 4.60, *p* = .002, partial *η* ^2^ = .005 (see **Figure 5**). The three-way interaction of sex, limbic disruption, and blocks was insignificant—*F* (3.44, 3,036.01) = 2.55, *p* = .046, partial *η* ^2^ = .003—and did not meet the adjusted alpha level for multiple comparisons (0.0125).

**Figure 6:**
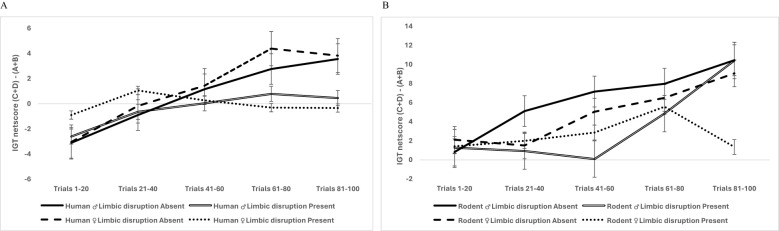
Data split by organism type showed that the interaction of sex, limbic perturbation, and IGT decision-making was not significant in humans **(A)**, but there was a significant interaction of sex and limbic disruption on the rodent’s block-wise decision-making, indicating that limbic perturbation impaired the rodent’s decision-making in a sex-specific manner where female rodents showed a prominent deficit in IGT decision-making **(B)**. The error bars show the standard error.

A follow-up analysis was done to understand the organism-specific effect of limbic disruption (limbic disruption was defined as vmPFC lesions in rodents and frontal and medial temporal lobe seizures in humans). We did data split by organism type and used a mixed analysis of variance for 2 sexes (male vs. female) × 2 limbic disruptions (absent vs. present) × 5 blocks of IGT (block 1, block 2, block 3, block 4, block 5) with block-wise net scores as the within-subject variable. Huynh–Feldt correction was applied, and the results for humans indicated that long-term decision-making improved across blocks: *F* (3.40, 2,440.99) = 18.51, *p* = .000, partial *η* ^2^ = .025, and age was significant: *F* (3.40, 2,440.99) = 7.28, *p* = .000, partial *η* ^2^ = .01. There was no effect of sex (*p* = .78). The effect of limbic disruption (frontal and medial temporal lobe epileptic seizures) was significant: *F* (3.40, 2,440.99) = 5.93, *p* = .000, partial *η* ^2^ = .008. The interaction effects of sex, limbic disruption, and blocks was non-significant (*p* = .46) (see **Figure 6A**). For rodents, long-term decision-making improved significantly across trials: *F* (3.51, 579.69) = 17.81, *p* = .000, partial *η* ^2^ = .097. The effect of age was not significant (*p* = .14). The effect of sex—*F* (3.51, 579.69) = 2.99, *p* = .023, partial *η* ^2^ =.018—did not meet the acceptable statistical level for multiple comparisons (0.0125). The limbic disruption (vmPFC lesions) had no effect, but the interaction of sex and limbic disruption on IGT blocks was significant: *F* (3.51, 579.69) = 3.58, *p* = .009, partial *η* ^2^ = .021. Female rodents with limbic disruption showed poor decision-making. The organism-specific results of limbic disruption indicated that limbic perturbation impaired decision-making (ventromedial prefrontal cortex in rodents and frontal/medial temporal lobe in humans). Female rodents showed the most adverse effects of limbic disruption (see **Figure 6B**). All of the effects that were significant at the.05 level survived the correction for multiple comparisons.

Lastly, we explored frequency-based choices or the deck B phenomenon (i.e., female participants prefer the risky short-term decks that give frequent rewards and infrequent punishments) associated with female IGT decision-making and is considered a risky choice per the somatic marker hypothesis. We examined the effect of organism type, sex, and CNS perturbation on frequency-based choices as deck type, i.e., infrequent punishment decks (deck B and deck D) versus frequent punishment decks (deck A and deck C). The results indicated a non-significant effect of deck type, as it did not meet the adjusted significance level for multiple comparisons: *F* (1, 883) = 5.34, *p* = .021, partial *η* ^2^ = .006. However, more choices were drawn from infrequent punishment decks B and D. Age had an insignificant effect on frequency-based deck choices: *F* (1, 883) = 4.49, *p* = .034, partial *η* ^2^ = .005, failing to meet the adjusted level of significance; organism type had a significant effect on deck choices: *F* (1, 883) = 47.29, *p* = .000, partial *η* ^2^ = .051, such that humans preferred infrequent punishment decks B and D (mean = 55.21) and avoided frequent punishment decks A and C (mean = 44.43). In contrast, the reverse was observed for rodents where decks A and C (mean = 39.19) were preferred over decks B and D (mean = 32.19). The effect of sex on deck choices was significant: *F* (1, 883) = 18.71, *p* = .000, partial *η* ^2^ = .021. Male rats preferred frequent punishment decks (means: decks B + D = 40.69; decks A + C = 44.33), whereas female rats preferred infrequent punishment decks (means: decks B + D = 46.70; decks A + C = 39.29). The choices of those with CNS perturbation differed from those of the healthy group: *F* (1, 883) = 7.71, *p* = .006, partial *η* ^2^ = .009, such that the healthy group preferred infrequent punishment decks (means: decks B + D = 47.82) over frequency punishment decks A + C = (mean = 42.40), whereas those with CNS perturbation showed reverse preference and made fewer choices that had infrequent punishments (means: decks B + D = 39.58) and more choices from frequent punishment decks A and C (mean = 41.22). The interaction effect of organism and sex was significant: *F* (1, 883) = 10.60, *p* = .001, partial *η* ^2^ = .012. Humans independent of sex preferred infrequent punishment decks more than frequent punishment decks (mean in male humans: decks B + D = 54.43; decks A + C = 45.03; mean in female humans: decks B + D = 55.98; decks A + C = 43.82), whereas in rodents, male–female rats differed in their choices: male rodents preferred frequent punishment decks and female rodents showed a reverse preference (mean for male rodents: decks B + D = 26.95; decks A + C = 43.62; mean for female rodents: decks B + D = 37.43; decks A + C = 34.77). The interaction of organism type and CNS perturbation was insignificant, *p* = .09. The interaction of sex type and CNS perturbation was significant: *F* (1, 883) = 6.79, *p* = .009, partial *η* ^2^ = .008. Healthy male participants preferred frequent punishments in both of the groups (healthy male mean decks B+D = 43.35 vs. decks A + C = 46.77; clinical male mean decks B + D = 38.03 vs. decks A + C = 41.88), whereas female participants in the healthy group preferred infrequent punishment decks (healthy female mean decks B + D = 52.29 vs. decks A + C = 38.03; the clinical group showed male mean decks B+D = 41.12 vs. decks A + C = 40.56). The interaction of organism, sex, and CNS perturbation did not meet the adjusted statistical level of significance for multiple comparisons: *F* (1, 883) = 5.09, *p* = .024, partial *η* ^2^ = .006. Although human decision-makers independent of sex and CNS perturbation preferred infrequent punishment decks, rodents preferred frequent punishment decks, except for female rats.

## 4 Discussion

Evolution preserves the molecular basis of behavioral circuits across species so that there is continuity in principles that govern basal to advance cognition associated with the complex nervous system (83), ensuring that cognitive processes such as assessing rewards and risks might be preserved across species with neural systems as varied as those of rodents and humans (84, 85). However, stark differences in cell, circuits, nervous systems, behavior, and the environment in which an organism operates make it difficult to draw useful insights from cross-species comparisons without rigorously drawn scientific hypotheses and robust experimental tasks. We relied on the SMH–IGT framework and tested if decision-making in humans and rodents is sensitive to psychological stress, CNS perturbation, limbic perturbation, and our results to discuss the effect of organism, age, and sex type on perturbations.

The analysis of psychological stress (stress in human hunger and rodent social isolation and CNS perturbations) and IGT decision-making showed that IGT performance improved across blocks. Age significantly affected the IGT, showing that old participants performed poorly compared to the young groups. The effect of psychological stress was significant; it impaired long-term decision-making; no other effects were significant. A follow-up analysis with data split by organism type was done to account for potential comparability in the stress experienced by humans (hunger) and rodents (social isolation). The results indicated that human task performance improved with trials, the age effect was significant, and stress impaired IGT decision-making only in humans. The rodents’ performance improved with trials. However, age, sex, or stress did not affect rodents’ IGT decision-making. We induced stress via the controlled duration of psychological constraints, namely, 3 h of timed food restriction before a big meal to evoke the feeling of hunger in humans and 6 h of maternal separation to evoke stress in rodents. The strength and novelty of this approach are that, unlike subjective and self-reported stress, we used controlled stress exposure, ensuring that stress exposure is measurable (in hours) and verifiable (treatment received). Our results showed that stress might impair long-term decision-making in humans, highlighting the possibility of stress as a psychological experience unique to human thought processes. Other studies have used social stress (Trier Social Stress Test: TSST) in humans and observed that anticipation of social evaluation increases psychophysiological stress and somatic response that lasts beyond 10 min after the stress has ceased (86) and is detrimental to IGT decision-making (34, 87–89). Even though psychological stress does not have localized CNS perturbation in the form of seizure, injury, or disease in the brain, it can impair cognitive functions such as decision-making in humans.

In contrast, rodents might be more resilient to stress effects. Contrary to our expectation, sex did not affect stress-related adverse effects on IGT decision-making. Some studies have reported no sex differences in how stress influences IGT (e.g., 88, 90). When stress failed to impact IGT decision-making adversely, the sample had more women than men (e.g., 91). The adverse effects of stress on male decision-making are attributed to the complex interaction of sex and stress hormones (89, 92). These remained unexamined in our study and could have impacted the results. Another reason might be that stress, anxiety, frustration, and sadness are considered negative emotional states, but their impact on decision-making could be variable. Trait anxiety (i.e., anxiety as a personality disposition rather than a temporary physiological state) impaired IGT performance but showed no sex differences (90), whereas frustration due to solving an unsolvable puzzle impaired male decision-making (36). Our results demonstrated that age had a significantly adverse effect on decision-making in humans. These results align with other reports where children perform worse than adolescents (93), and age effect is curvilinear such that decision-making improves in late adolescence due to changes in striatal versus frontal circuitry (94) but declines in old age (93). The task might offer a way to delineate aging-related cognitive deficits distinct from executive functions that rely on the staggered prefrontal cortex development (see 95, 96). Our results indicate that IGT performance is sensitive to old age. The IGT might be useful for delineating age-specific structural and functional changes due to aging. Furthermore, the IGT with other tasks of spatial working memory in humans and rodents (e.g., trail making, visual cue learning, object recognition) or impulsivity, inhibition, and motor control might help identify signs of CNS disintegration at an early age. Our results also demonstrated that animal models of stress could elicit resilience to early-life social isolation. Studies of hypothalamic–pituitary–adrenal axis (HPA) and stress-diathesis models for brain and cognitive disorders should consider potentially greater resilience in animal model systems compared to higher vulnerability in humans.

Our results examining the effect of CNS perturbation showed a significant main effect of the IGT blocks, indicating that long-term decision-making improved in humans and rodents across 100 trials; both learned to differentiate between risky, short-term versus safe, long-term rewards and shifted their preference to the latter as the task progressed. Our results indicated that the effect of organism type did not hold against the adjusted level of statistical significance, indicating that the difference between human and rodent long-term decision-making was not statistically different. Only one study compared human and rodent decision-making; our results align with their results (23). Others have also observed that mice and rats perform the IGT similarly to humans and report that rodents are faster learners (97, 98). We observed that CNS perturbation adversely affected IGT decision-making (human participants with a neurological or neuropsychiatric disorder of epilepsy, spinal injury, and major depressive disorder and rodents with lesions made short-term risky choices from decks A and B) compared to the healthy group. The interaction of organism type and CNS perturbation was significant; humans diagnosed with clinical disorders (CNS perturbation in seizure, injury, lesion, and depression) performed poorly on the task compared to rodents with CNS perturbation (lesions). The results align with human studies where poor long-term decision-making is observed in neurological and neuropsychiatric conditions (37–39, 44, 66, 67). The task showed sensitivity to select neurological and psychiatric disorders and could be a valuable tool to assess decision-making deficits due to CNS perturbation in humans and rodents (i.e., epileptic brain seizures, spinal cord injury, major depressive disorder in humans, and brain lesions in rodents). These results might be helpful for rodent studies of neuropathology aimed at identifying specific genes, neurotransmitters (e.g., serotonin-dopaminergic pathways), and neural circuits (cortico-limbic) as potential targets for pharmaceutical interventions for disorders involving rewards, impulsivity, and maladaptive decision-making.

The results from the analysis of limbic perturbation (prefrontal cortex and medial temporal lobe structures) on IGT showed the main effect of the IGT blocks, indicating that IGT improved with trials, the effect of age was significant, and the presence of limbic perturbation was detrimental to long-term decision-making in the IGT. Our results align with the SMH–IGT assumption and demonstrate that limbic disruption (ventromedial prefrontal cortex lesions in rodents and frontal or medial temporal lobe seizures in humans) adversely impacted IGT decision-making. A follow-up analysis was done to examine organism-specific effects of limbic perturbation. The results for humans indicated that performance improved with trials, but old age and limbic perturbation impaired human IGT decision-making. For rodents, performance improved with trials, and the effect of sex did not meet the adjusted level of significance, but interaction of sex and limbic perturbation significantly impacted IGT such that female rodent decision-making showed the most detrimental effect of limbic perturbation. Our results from a rodent–human comparison suggest that damage to the limbic system might have sex-specific adverse effects in rodents, especially when the ventromedial prefrontal cortex is damaged, and age-specific adverse effects in humans when the frontal and medial temporal regions are affected. Earlier IGT studies showed double dissociation such that ventromedial prefrontal cortex (vmPFC) damage produced a prominent IGT deficit compared to amygdala damage (56). Future cross-species studies should compare the role of vmPFC damage in rodents and humans. Interestingly, sex did not influence IGT when examined under psychological stress, and in the case of CNS perturbation, organism-specific analysis showed sex-specific effects of limbic perturbations in rodents.

Additional results from the analysis of frequency deck choices indicated that deck type (frequent vs. infrequent punishment decks) and age did not meet the adjusted significance level. The effect of the organism (more humans versus rodents preferred infrequent punishment decks B & D), sex (more female versus male participants preferred decks B & D), and CNS perturbation were significant (the healthy group selected more options with infrequent punishment decks B & D). The interaction of sex and organism type had a significant effect on frequency-based choices, indicating that frequent-based choices in humans showed no sex differences, but sex differences were prominent in rodents, where female rodents preferred infrequent punishments. The interaction of sex and CNS perturbation was significant; irrespective of clinical group, male participants preferred frequent punishments, but female participants preferred infrequent punishment. The results provide robust evidence for the “deck B phenomenon” where female participants prefer decks with infrequent punishment (decks B and D) over frequent punishment decks (decks A and C), prominently in rodents. The results demonstrated that CNS perturbations were associated with frequent punishment deck selections: the healthy group preferred infrequent punishment decks. Our results align with those of others and demonstrated the female participants’ preference for infrequent punishments (27); however, we might be the first to observe that preference for infrequent punishment is associated with the healthy group rather than the CNS perturbed clinical group. It provides a reason for investigating deck B preference and examining the extent to which it might be preserved across species as different as rodents and humans for possible sex-specific cross-species comparisons.

Overall, our results examined cross-species decision-making in the IGT. Our results demonstrated that CNS perturbation, limbic loop perturbation, and psychological stress adversely impact long-term decision-making. We demonstrated that limbic perturbation of ventromedial prefrontal (vmPFC) in rodents has sex-specific deficits, and disruption in the frontal and medial temporal (MTL) regions in humans has an age-specific deficit in the task. Furthermore, our results demonstrated that infrequent punishment decks are preferred by humans more than rodents, by the healthy group more than those with CNS perturbation, and by female participants more than male decision-makers. Our results were corrected for multiple comparisons and presented a rigorous way to test the SMH–IGT assumptions about long-term decision-making. Our findings offer insights for translational neuroscience, where accurate measures of cognitive deficits in animal models might help understand stress vulnerability in neurological and psychiatric disorders. Delineating the nature, duration, and onset of stress is important for understanding the effect of stress on the prefrontal cortex network (99) and how it amplifies the effects of disorders such as post-stress traumatic disorder, depression, and anxiety (100–102). These results add insights to IGT as a useful cross-species circuitry, such as prefrontal regions and medial temporal regions such as the amygdala and the hippocampus, and a measure of risk and reward impulsivity, cognitive control, and reward learning across trials and memory systems. Creating a comparable task behavior might improve the modeling of cognitive, affective, and behavioral deficits in disorders such as addiction, gambling, and eating disorders, where rewards such as drugs, money, and food can be made comparable across species. Such endeavors will improve our understanding of the cognitive consequences of neurological and psychiatric disorders. A comparison of human and rodent choice behavior in health and disease conditions is required for translational research.

Notwithstanding the strengths listed above, the study has several limitations, a few of which are listed below. Our pooled data with gender/sex imbalance might have failed to fully capture sex differences in the effect of stress on decision-making, except in the case of limbic disruption in rodents. There were fewer female rodents and fewer women in the clinical group of CNS perturbation, the spinal injury sample in the human data set was male-dominated (a significant cause of spinal injury is driving/road accidents; male drivers are more than female drivers), and the majority of female participants in our studies were engineering students and might be less representative of female decision-makers as they are selected based on a highly competitive exam that relies on risk-taking abilities. Furthermore, the clinical group of CNS perturbation had human participants diagnosed with neurological or psychiatric disorders (epilepsy, spinal injury, major depressive disorder) and rodents with lesions; we did not control for the duration of conditions in humans and rodents, and we would expect that future studies of human–rodent comparison would control the onset, severity, and duration of clinical disorders, making species-specific adjustments for age. Similarly, a small-sample-sized animal dataset might have influenced the results. However, we tried addressing this concern by splitting data by organism type, ensuring the effect of stress on decision-making factors in species-specific stress. More variations in the IGT structure need to be tested to establish parity between rodent and human tasks through repeated human–rodent comparisons. Furthermore, it is difficult to identify factors that evoke cognitive and behavioral responses in species-specific but equitable ways and influence experimental tasks—for instance, money is used for the human version of the IGT, a secondary reinforcer, whereas food is used for rodent versions of the task and is a primary reinforcer. Fewer studies have examined human and rodent task performance. It is unclear if species-specific changes in the animal version of the task, especially using food instead of monetary rewards (23), elicit risk, rewards, and long-term decision-making (24, 25). Furthermore, deck choices, reaction time, and latencies are crucial for understanding the cognitive processing involved in decision-making, which we did not use. We also could not account for variability in the pre-testing environment across studies used to pool the data and did not control for all factors such as body mass index (BMI) in humans when we controlled for the weight of rodents for homogeneity within species. Other factors such as reward sensitivity, anxiety, and working memory capacity were not measured but would improve the comparability between human and rodent decision-making models. Lastly, we acknowledge that it is challenging to create precisely equitable rodent–human stress conditions; specific parameters such as non-localized CNS alteration indicative of the nature, severity, and extent of interference with somatic cues might offer objective criteria for comparing animal–human models of cognitive deficits. The stress of social exclusion via maternal separation in rodents and experiencing hunger via food restriction in humans are not equitable, but both involve non-localized, non-pathological temporary alteration of the nervous system that is distinct from physiological response due to brain lesion, injury, or surgery; both might alter the sensitivity to rewards, learning, memory, and motivation systems and hence can impair long-term decision-making.

The previous studies of clinical disorders and CNS perturbations showed IGT deficits when pathophysiological alteration was restricted to the brain or the spinal cord; however, brain perturbations impact the cognitive system, and spinal perturbations impact the sensory and motor system. Our study allowed us to demonstrate that perturbations to the CNS comprising the brain and spinal cord impair IGT decision. The IGT might serve as a good cross-species measure of neural systems that contribute to cognitive, emotional, and motor aspects of decision-making function that relies on integrated sensory, motor, and cognitive information processing. Secondly, we demonstrated that psychological stress adversely impacts human decision-making; the rodent–human studies of the hypothalamus–pituitary–adrenal (HPA) axis and clinical disorders might find these results helpful (103). The findings of the sex-specific adverse effect of limbic perturbation add insights into sexual dimorphism of brain structure and function with potential clinical implications for neurological and neuropsychiatric disorders (104, 105). Furthermore, there are several potential benefits from these insights: studying the effects of age on decision-making in real life has limitations, such that young children are not permitted to make decisions that involve risks (93, 106). Creating specific neuropsychiatric conditions such as “depression” in rodents poses a risk of anthropomorphizing, and there are species-specific limitations on creating neuropsychiatric symptoms such as hallucinations in animals. Therefore, experimental tasks that create discrete and measurable behavior help understand cognitive deficits in clinical disorders (107–109). These insights will be helpful for preclinical and clinical studies in translational neuroscience research. Sex differences have recently attracted attention in neurobiology and brain sciences (110, 111), and IGT studies show sex differences that are puzzling because women are risk averse in real life but take high risks in the IGT (65). Future studies should investigate possible female resilience to stress when it is detrimental to male decision-making (27, 34, 87–89). Interdisciplinary neurobiology and cognitive science research might reduce disease burden by improving early detection, diagnosis, and treatment. The IGT bridged the translational gap between animal and human studies and served as a low-cost behavioral assay for reliable cross-species comparisons of causal mechanisms in clinical disorders. Species-specific changes in the animal version of the IGT and similar tasks allow us to examine if using food instead of monetary rewards evokes risk, rewards, and long-term decision-making in a comparable manner (see 24, 25). Our work focused on the IGT; however, comparisons of other tasks, such as the Balloon Analog Risk Task (BART) and Delayed Discounting Task (DDT), would help us understand animal cognition and decision-making under stress and trauma, improving cross-species structure–function mapping for brain cognition studies (112). Previously, we documented that the Theory of Mind cognition is sensitive to lateralized limbic perturbation of medial temporal structures (amygdala and hippocampus) (113). A cross-species comparison of the Theory of Mind provides insights into shared mental representation and cognitive abilities that might diverge through evolutionary mechanisms shaping the phylogenetic tree.

## Data Availability

The raw data supporting the conclusions of this article will be made available by the authors, without undue reservation.

## Appendix A

**Table A:** Rodent-human comparison of decision making in the Iowa Gambling Task under psychological stress and CNS perturbation showed key results such as : (a) healthy vs. stress comparison showed old age and stress impaired IGT in humans (b) healthy vs. CNS perturbation comparison showed CNS perturbation impaired IGT decision making, rodents showed greater resilience than humans (c) limbic perturbation impaired IGT decision making, effect might be age-specific in humans and sex-specific in rodents.

R O D E N T IGT	Healtdy Group	Psychological stress: social exclusion induced (6 hrs isolation)	CNS perturbed: sham lesioned group/non-task specific lesion	CNS perturbed: stress + sham lesioned group	CNS perturbed: task-specific lesion group (vmPFC)	CNS perturbed: stress+ task specific lesion group (vmPFC)
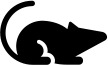
Comparisons		Stressed				
			CNS Perturbation			
					Limbic Perturbation	
Iowa Gambling Task by Healthy, Stressed, CNS perturbed, Limbic loop perturbed groups						

H U M A N IGT	Healtdy Group	Psychological stress: hunger induced (3 hrs food restriction)	CNS perturbed: clinical group Major Depressive Disorder	CNS perturbed: clinical group of Spinal cord injury (1 yr cervical injury)	CNS perturbed: clinical group focal epilepsy seizures (frontal, MTL)	CNS perturbed: clinical group of drug-resistant epilepsy + epilepsy surgery (frontal, medial temporal lobe)
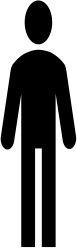
Comparisons		Stressed				
			CNS Perturbation			
					Limbic perturbation	

## References

[1] Barron HC Mars RB Dupret D Lerch JP Sampaio-Baptista C. Cross-species neuroscience: closing the explanatory gap. Philos Trans R Soc B Biol Sci. (2021) 376:20190633. doi: 10.1098/rstb.2019.0633, PMID: 33190601 PMC7116399

[2] Bechara A Damasio AR Damasio H Anderson SW. Insensitivity to future consequences following damage to human prefrontal cortex. Cognition. (1994) 50:7–15. doi: 10.1016/0010-0277(94)90018-3, PMID: 8039375

[3] Damasio AR. The somatic marker hypothesis and the possible functions of the prefrontal cortex. Philos Trans R Soc Lond B Biol Sci. (1996) 351(1346):1413–20. doi: 10.1098/rstb.1996.0125, PMID: 8941953

[4] Dunn BD Dalgleish T Lawrence AD. The somatic marker hypothesis: A critical evaluation. Neurosci Biobehav Rev. (2006) 30:239–71. doi: 10.1016/j.neubiorev.2005.07.001, PMID: 16197997

[5] Aram S Levy L Patel JB Anderson AA Zaragoza R Dashtestani H. The Iowa Gambling Task: A review of the historical evolution, scientific basis, and use in functional neuroimaging. SAGE Open. (2019) 9:215824401985691. doi: 10.1177/2158244019856911

[6] Bechara A Damasio H Damasio AR Lee GP. Different contributions of the human amygdala and ventromedial prefrontal cortex to decision-making. J Neurosci. (1999) 19:5473–81. doi: 10.1523/JNEUROSCI.19-13-05473.1999, PMID: 10377356 PMC6782338

[7] Brand M Grabenhorst F Starcke K Vandekerckhove MMP Markowitsch HJ. Role of the amygdala in decisions under ambiguity and decisions under risk: evidence from patients with Urbach-Wiethe disease. Neuropsychologia. (2007) 45:1305–17. doi: 10.1016/j.neuropsychologia.2006.09.021, PMID: 17070876

[8] Gupta R Duff MC Denburg NL Cohen NJ Bechara A Tranel D. Declarative memory is critical for sustained advantageous complex decision-making. Neuropsychologia. (2009) 47:1686–93. doi: 10.1016/j.neuropsychologia.2009.02.007, PMID: 19397863 PMC2697903

[9] Thiel A Hilker R Kessler J Habedank B Herholz K Heiss W-D. Activation of basal ganglia loops in idiopathic Parkinson’s disease: a PET study. J Neural Transm Vienna Austria 1996. (2003) 110:1289–301. doi: 10.1007/s00702-003-0041-7, PMID: 14628193

[10] Colautti L Iannello P Silveri MC Antonietti A. Decision-making under ambiguity and risk and executive functions in Parkinson’s disease patients: A scoping review of the studies investigating the Iowa Gambling Task and the Game of Dice. Cogn Affect Behav Neurosci. (2023) 23:1225–43. doi: 10.3758/s13415-023-01106-3, PMID: 37198383 PMC10545597

[11] Cardoso CdO Branco LD Cotrena C Kristensen CH Schneider Bakos DDG Fonseca RP. The impact of frontal and cerebellar lesions on decision making: evidence from the Iowa Gambling Task. Front Neurosci. (2014) 8:61. doi: 10.3389/fnins.2014.00061, PMID: 24782697 PMC3986592

[12] Grone BP Baraban SC. Animal models in epilepsy research: legacies and new directions. Nat Neurosci. (2015) 18:339–43. doi: 10.1038/nn.3934, PMID: 25710835

[13] Stout JC Busemeyer JR Lin A Grant SJ Bonson KR. Cognitive modeling analysis of decision-making processes in cocaine abusers. Psychon Bull Rev. (2004) 11:742–7. doi: 10.3758/BF03196629, PMID: 15581127

[14] Verdejo-Garcia A Bechara A Recknor EC Perez-Garcia M. Decision-making and the Iowa Gambling Task: Ecological validity in individuals with substance dependence. Psychol Belgica. (2006) 46:55–78. doi: 10.5334/pb-46-1-2-55

[15] Verdejo-Garcia A Benbrook A Funderburk F David P Cadet J-L Bolla KI. The differential relationship between cocaine use and marijuana use on decision-making performance over repeat testing with the Iowa Gambling Task. Drug Alcohol Depend. (2007) 90:2–11. doi: 10.1016/j.drugalcdep.2007.02.004, PMID: 17367959 PMC1986840

[16] Stevens L Betanzos-Espinosa P Crunelle CL Vergara-Moragues E Roeyers H Lozano O. Disadvantageous decision-making as a predictor of drop-out among cocaine-dependent individuals in long-term residential treatment. Front Psychiatry. (2013) 4:149. doi: 10.3389/fpsyt.2013.00149, PMID: 24298260 PMC3828507

[17] Verdejo-García A Lawrence AJ Clark L. Impulsivity as a vulnerability marker for substance-use disorders: review of findings from high-risk research, problem gamblers and genetic association studies. Neurosci Biobehav Rev. (2008) 32:777–810. doi: 10.1016/j.neubiorev.2007.11.003, PMID: 18295884

[18] Rosen HJ Levenson RW. The emotional brain: Combining insights from patients and basic science. Neurocase. (2009) 15:173–81. doi: 10.1080/13554790902796787, PMID: 20183547 PMC2917380

[19] Singh V. Bittersweet memories and somatic marker hypothesis: adaptive control in emotional recall facilitates long-term decision-making in the Iowa Gambling Task. Front Neurosci. (2024) 17:1214271. doi: 10.3389/fnins.2023.1214271, PMID: 38292897 PMC10824841

[20] Singh V. A potential role of reward and punishment in the facilitation of the emotion-cognition dichotomy in the Iowa Gambling Task. Front Psychol. (2013) 4:944. doi: 10.3389/fpsyg.2013.00944, PMID: 24381567 PMC3865383

[21] Lin C-H Chiu Y-C Lee P-L Hsieh J-C. Is deck B a disadvantageous deck in the Iowa Gambling Task? Behav. Brain Funct. (2007) 3:16. doi: 10.1186/1744-9081-3-16, PMID: 17362508 PMC1839101

[22] Manes F Sahakian B Clark L Rogers R Antoun N Aitken M. Decision-making processes following damage to the prefrontal cortex. Brain. (2002) 125:624–39. doi: 10.1093/brain/awf049, PMID: 11872618

[23] Cabeza L Giustiniani J Chabin T Ramadan B Joucla C Nicolier M. Modelling decision-making under uncertainty: A direct comparison study between human and mouse gambling data. Eur Neuropsychopharmacol J Eur Coll Neuropsychopharmacol. (2020) 31:58–68. doi: 10.1016/j.euroneuro.2019.11.005, PMID: 31837913

[24] Van den Bos R Lasthuis W den Heijer E van der Harst J Spruijt B. Toward a rodent model of the Iowa Gambling Task. Behav Res Methods. (2006) 38:470–8. doi: 10.3758/BF03192801, PMID: 17186757

[25] de Visser L Homberg J Mitsogiannis M Zeeb F Rivalan M Fitoussi A. Rodent versions of the Iowa Gambling Task: opportunities and challenges for the understanding of decision-making. Front Neurosci. (2011) 5:109. doi: 10.3389/fnins.2011.00109, PMID: 22013406 PMC3189637

[26] Rivalan M Coutureau E Fitoussi A Dellu-Hagedorn F. Inter-individual decision-making differences in the effects of cingulate, orbitofrontal, and prelimbic cortex lesions in a rat gambling task. Front Behav Neurosci. (2011) 5:22. doi: 10.3389/fnbeh.2011.00022, PMID: 21559308 PMC3085860

[27] van den Bos R Homberg J de Visser L. A critical review of sex differences in decision-making tasks: Focus on the Iowa Gambling Task. Behav Brain Res. (2013) 238:95–108. doi: 10.1016/J.BBR.2012.10.002, PMID: 23078950

[28] Van Den Bos R Koot S de Visser L. A rodent version of the Iowa Gambling Task: 7 years of progress. Front Psychol. (2014) 5:203. doi: 10.3389/fpsyg.2014.00203, PMID: 24672498 PMC3957418

[29] Li X Lu ZL D’Argembeau A Ng M Bechara A. The Iowa Gambling Task in fMRI images. Hum Brain Mapp. (2010) 31:410–23. doi: 10.1002/hbm.20875, PMID: 19777556 PMC2826566

[30] Lupien SJ McEwen BS Gunnar MR Heim C. Effects of stress throughout the lifespan on the brain, behaviour and cognition. Nat Rev Neurosci. (2009) 10:434–45. doi: 10.1038/nrn2639, PMID: 19401723

[31] Sandi C Haller J. Stress and the social brain: behavioural effects and neurobiological mechanisms. Nat Rev Neurosci. (2015) 16:290–304. doi: 10.1038/nrn3918, PMID: 25891510

[32] Kogler L Müller VI Chang A Eickhoff SB Fox PT Gur RC. Psychosocial versus physiological stress—Meta-analyses on deactivations and activations of the neural correlates of stress reactions. Neuroimage. (2015) 119:235–51. doi: 10.1016/j.neuroimage.2015.06.059, PMID: 26123376 PMC4564342

[33] Witbracht MG Laugero KD Van Loan MD Adams SH Keim NL. Performance on the Iowa Gambling Task is related to magnitude of weight loss and salivary cortisol in a diet-induced weight loss intervention in overweight women. Physiol Behav. (2012) 106:291–7. doi: 10.1016/j.physbeh.2011.04.035, PMID: 21565212

[34] Preston SD Buchanan TW Stansfield RB Bechara A. Effects of anticipatory stress on decision making in a gambling task. Behav Neurosci. (2007) 121:257–63. doi: 10.1037/0735-7044.121.2.257, PMID: 17469915

[35] Van den Bos R Harteveld M Stoop H. Stress and decision-making in humans: performance is related to cortisol reactivity, albeit differently in men and women. Psychoneuroendocrinology. (2009) 34:1449–58. doi: 10.1016/j.psyneuen.2009.04.016, PMID: 19497677

[36] Starcke K Agorku JD Brand M. Exposure to unsolvable anagrams impairs performance on the Iowa Gambling Task. Front Behav Neurosci. (2017) 11:114. doi: 10.3389/fnbeh.2017.00114, PMID: 28642693 PMC5462929

[37] Levine B Black SE Cheung G Campbell A O’Toole C Schwartz ML. Gambling task performance in traumatic brain injury: relationships to injury severity, atrophy, lesion location, and cognitive and psychosocial outcome. Cogn Behav Neurol. (2005) 18:45. doi: 10.1097/01.wnn.0000152227.13001.c3, PMID: 15761276

[38] Fujiwara E Schwartz ML Gao F Black SE Levine B. Ventral frontal cortex functions and quantified MRI in traumatic brain injury. Neuropsychologia. (2008) 46:461–74. doi: 10.1016/j.neuropsychologia.2007.08.027, PMID: 17976665 PMC2287189

[39] Cogo MG Rota S Fusco ML Mapelli C Ferri F Appollonio IM. Cognitive correlates of under-ambiguity and under-risk decision making in high-functioning patients with relapsing remitting multiple sclerosis. J Clin Exp Neuropsychol. (2014) 36:1066–75. doi: 10.1080/13803395.2014.971718, PMID: 25486588

[40] Labudda K Frigge K Horstmann S Aengenendt J Woermann FG Ebner A. Decision making in patients with temporal lobe epilepsy. Neuropsychologia. (2009) 47:50–8. doi: 10.1016/j.neuropsychologia.2008.08.014, PMID: 18789345

[41] MacPherson SE Phillips LH Della Sala S Cantagallo A. Iowa Gambling Task impairment is not specific to ventromedial prefrontal lesions. Clin Neuropsychol. (2009) 23:510–22. doi: 10.1080/13854040802396586, PMID: 18979282

[42] Bechara A Tranel D Damasio H. Characterization of the decision-making deficit of patients with ventromedial prefrontal cortex lesions. Brain. (2000) 123:2189–202. doi: 10.1093/BRAIN/123.11.2189, PMID: 11050020

[43] Clark L Manes F Antoun N Sahakian BJ Robbins TW. The contributions of lesion laterality and lesion volume to decision-making impairment following frontal lobe damage. Neuropsychologia. (2003) 41:1474–83. doi: 10.1016/S0028-3932(03)00081-2, PMID: 12849765

[44] Fellows LK Farah MJ. Different underlying impairments in decision-making following ventromedial and dorsolateral frontal lobe damage in humans. Cereb Cortex. (2005) 15:58–63. doi: 10.1093/cercor/bhh108, PMID: 15217900

[45] North NT O’Carroll RE. Decision making in patients with spinal cord damage: afferent feedback and the somatic marker hypothesis. Neuropsychologia. (2001) 39:521–4. doi: 10.1016/s0028-3932(00)00107-x, PMID: 11254934

[46] Nardone R Höller Y Brigo F Seidl M Christova M Bergmann J. Functional brain reorganization after spinal cord injury: systematic review of animal and human studies. Brain Res. (2013) 1504:58–73. doi: 10.1016/j.brainres.2012.12.034, PMID: 23396112

[47] Singh V Khan A. Decision making in the reward and punishment variants of the Iowa Gambling Task: evidence of “foresight” or “framing”? Front Neurosci. (2012) 6:107. doi: 10.3389/fnins.2012.00107, PMID: 22833714 PMC3400253

[48] Engel J Jr. Mesial temporal lobe epilepsy: what have we learned? Neuroscientist. (2001) 7:340–52. doi: 10.1177/107385840100700410, PMID: 11488399

[49] Simsekoglu R Tombul T Demirci H Özdemir M Ankaralı H. Comparison of decision-making under ambiguity in patients with temporal lobe and frontal lobe epilepsy. Epilepsy Behav. (2022) 129:108636. doi: 10.1016/j.yebeh.2022.108636, PMID: 35259626

[50] Yamano M Akamatsu N Tsuji S Kobayakawa M Kawamura M. Decision-making in temporal lobe epilepsy examined with the Iowa Gambling Task. Epilepsy Res. (2011) 93:33–8. doi: 10.1016/j.eplepsyres.2010.10.009, PMID: 21106350

[51] Zhang L Qiu X Zhu X Zou X Chen L. Decision-making in patients with epilepsy: A systematic review and meta-analysis. Epilepsy Res. (2018) 148:55–62. doi: 10.1016/j.eplepsyres.2018.10.009, PMID: 30384115

[52] Fernando ABP Robbins TW . Animal models of neuropsychiatric disorders. Annu Rev Clin Psychol. (2011) 7:39–61. doi: 10.1146/annurev-clinpsy-032210-104454, PMID: 21219191

[53] Murray EA Wise SP Drevets WC. Localization of dysfunction in major depressive disorder: prefrontal cortex and amygdala. Biol Psychiatry. (2011) 69:e43–54. doi: 10.1016/j.biopsych.2010.09.041, PMID: 21111403 PMC3058124

[54] Price JL Drevets WC. Neural circuits underlying the pathophysiology of mood disorders. Trends Cogn Sci. (2012) 16:61–71. doi: 10.1016/j.tics.2011.12.011, PMID: 22197477

[55] Rolls ET. A non-reward attractor theory of depression. Neurosci Biobehav Rev. (2016) 68:47–58. doi: 10.1016/j.neubiorev.2016.05.007, PMID: 27181908

[56] Bechara A Damasio H Tranel D Damasio AR. Deciding advantageously before knowing the advantageous strategy. Science. (1997) 275:1293–5. doi: 10.1126/science.275.5304.1293, PMID: 9036851

[57] Roxo MR Franceschini PR Zubaran C Kleber FD Sander JW. The limbic system conception and its historical evolution. Sci World J. (2011) 11:2427–40. doi: 10.1100/2011/157150, PMID: 22194673 PMC3236374

[58] Yavas E Gonzalez S Fanselow MS. Interactions between the hippocampus, prefrontal cortex, and amygdala support complex learning and memory. F1000Research. (2019) 8:F1000–Faculty. doi: 10.12688/f1000research, PMID: 31448084 PMC6676505

[59] Kovács I Richman MJ Janka Z Maraz A Andó B. Decision making measured by the Iowa Gambling Task in alcohol use disorder and gambling disorder: a systematic review and meta-analysis. Drug Alcohol Depend. (2017) 181:152–61. doi: 10.1016/j.drugalcdep.2017.09.023, PMID: 29055269

[60] Shurman B Horan WP Nuechterlein KH. Schizophrenia patients demonstrate a distinctive pattern of decision-making impairment on the Iowa Gambling Task. Schizophr Res. (2005) 72:215–24. doi: 10.1016/j.schres.2004.03.020, PMID: 15560966

[61] Sevy S Burdick KE Visweswaraiah H Abdelmessih S Lukin M Yechiam E. Iowa Gambling Task in schizophrenia: A review and new data in patients with schizophrenia and co-occurring cannabis use disorders. Schizophr Res. (2007) 92:74–84. doi: 10.1016/j.schres.2007.01.005, PMID: 17379482 PMC2039912

[62] Cavedini P Riboldi G D’Annucci A Belotti P Cisima M Bellodi L. Decision-making heterogeneity in obsessive-compulsive disorder: ventromedial prefrontal cortex function predicts different treatment outcomes. Neuropsychologia. (2002) 40:205–11. doi: 10.1016/s0028-3932(01)00077-x, PMID: 11640942

[63] da Rocha FF Alvarenga NB Malloy-Diniz L Corrêa H. Decision-making impairment in obsessive-compulsive disorder as measured by the Iowa Gambling Task. Arq Neuropsiquiatr. (2011) 69:642–7. doi: 10.1590/s0004-282x2011000500013, PMID: 21877034

[64] Krishna R Udupa S George CM Kumar KJ Viswanath B Kandavel T. Neuropsychological performance in OCD: a study in medication-naïve patients. Prog Neuropsychopharmacol Biol Psychiatry. (2011) 35:1969–76. doi: 10.1016/j.pnpbp.2011.09.009, PMID: 21967733

[65] Singh V Thakral S Singh K Garg R. Examining cognitive sex differences in elite math intensive education: Preliminary evidence from a gender inequitable country. Trends Neurosci Educ. (2022) 26:100172. doi: 10.1016/j.tine.2022.100172, PMID: 35303976

[66] Must A Szabó Z Bódi N Szász A Janka Z Kéri S. Sensitivity to reward and punishment and the prefrontal cortex in major depression. J Affect Disord. (2006) 90:209–15. doi: 10.1016/j.jad.2005.12.005, PMID: 16412520

[67] Must A Horvath S Nemeth VL Janka Z. The Iowa Gambling Task in depression – what have we learned about sub-optimal decision-making strategies? Front Psychol. (2013) 4:732. doi: 10.3389/fpsyg.2013.00732, PMID: 24133474 PMC3794198

[68] Wang Y Chen Q Zhang X Wang K Cheng H Chen X. Changes in decision-making function in patients with subacute mild traumatic brain injury. Eur J Neurosci. (2024) 59:69–81. doi: 10.1111/ejn.16195, PMID: 38044718

[69] Mueller EM Nguyen J Ray WJ Borkovec TD. Future-oriented decision-making in Generalized Anxiety Disorder is evident across different versions of the Iowa Gambling Task. J Behav Ther Exp Psychiatry. (2010) 41:165–71. doi: 10.1016/j.jbtep.2009.12.002, PMID: 20060098

[70] Werner NS Duschek S Schandry R. Relationships between affective states and decision-making. Int J Psychophysiol. (2009) 74:259–65. doi: 10.1016/j.ijpsycho.2009.09.010, PMID: 19808059

[71] Brevers D Bechara A Cleeremans A Noël X. Iowa Gambling Task (IGT): twenty years after – gambling disorder and IGT. Front Psychol. (2013) 4:665. doi: 10.3389/fpsyg.2013.00665, PMID: 24137138 PMC3786255

[72] Vassileva J Petkova P Georgiev S Martin EM Tersiyski R Raycheva M. Impaired decision-making in psychopathic heroin addicts. Drug Alcohol Depend. (2007) 86:287–9. doi: 10.1016/j.drugalcdep.2006.06.015, PMID: 16930861

[73] Barry D Petry NM. Predictors of decision-making on the Iowa Gambling Task: Independent effects of lifetime history of substance use disorders and performance on the Trail Making Test. Brain Cogn. (2008) 66:243–52. doi: 10.1016/j.bandc.2007.09.001, PMID: 17942206 PMC2292486

[74] Frank MJ Seeberger LC O’Reilly RC. By carrot or by stick: cognitive reinforcement learning in Parkinsonism. Science. (2004) 306:1940–3. doi: 10.1126/science.1102941, PMID: 15528409

[75] Jacus J-P Gély-Nargeot M-C Bayard S. Ecological relevance of the Iowa Gambling Task in patients with Alzheimer’s disease and mild cognitive impairment. Rev Neurol (Paris). (2018) 174:327–36. doi: 10.1016/j.neurol.2017.08.003, PMID: 29706297

[76] Denburg NL Tranel D Bechara A. The ability to decide advantageously declines prematurely in some normal older persons. Neuropsychologia. (2005) 43:1099–106. doi: 10.1016/j.neuropsychologia.2004.09.012, PMID: 15769495

[77] Kobayakawa M Koyama S Mimura M Kawamura M. Decision making in Parkinson’s disease: Analysis of behavioral and physiological patterns in the Iowa Gambling Task. Mov Disord Off J Mov Disord Soc. (2008) 23:547–52. doi: 10.1002/mds.21865, PMID: 18069681

[78] Cavanagh JF Gregg D Light GA Olguin SL Sharp RF Bismark AW. Electrophysiological biomarkers of behavioral dimensions from cross-species paradigms. Trans Psychiatry. (2021) 11:482. doi: 10.1038/s41398-021-01562-w, PMID: 34535625 PMC8448772

[79] Potenza MN. The importance of animal models of decision making, gambling, and related behaviors: implications for translational research in addiction. Neuropsychopharmacology. (2009) 34:2623–4. doi: 10.1038/npp.2009.152, PMID: 19901921 PMC2871541

[80] Sachdev PS Blacker D Blazer DG Ganguli M Jeste DV Paulsen JS. Classifying neurocognitive disorders: the DSM-5 approach. Nat Rev Neurol. (2014) 10:634–42. doi: 10.1038/nrneurol.2014.181, PMID: 25266297

[81] Flint A Raben A Blundell JE Astrup A. Reproducibility, power and validity of visual analogue scales in assessment of appetite sensations in single test meal studies. Int J Obes. (2000) 24:38–48. doi: 10.1038/sj.ijo.0801083, PMID: 10702749

[82] Singh V Schiebener J Müller SM Liebherr M Brand M Buelow MT. Country and sex differences in decision making under uncertainty and risk. Front Psychol. (2020) 11:486. doi: 10.3389/fpsyg.2020.00486, PMID: 32265793 PMC7101158

[83] Lyon P Keijzer F Arendt D Levin M. Reframing cognition: getting down to biological basics. Philos Trans R Soc B. (2021) 376:20190750. doi: 10.1098/rstb.2019.0750, PMID: 33487107 PMC7935032

[84] van der Staay FJ. Animal models of behavioral dysfunctions: basic concepts and classifications, and an evaluation strategy. Brain Res Rev. (2006) 52:131–59. doi: 10.1016/j.brainresrev.2006.01.006, PMID: 16529820

[85] Fuccillo MV Rothwell PE Malenka RC. From synapses to behavior: what rodent models can tell us about neuropsychiatric disease. Biol Psychiatry. (2016) 79:4–6. doi: 10.1016/j.biopsych.2015.02.009, PMID: 26616432 PMC4832414

[86] Het S Rohleder N Schoofs D Kirschbaum C Wolf OT. Neuroendocrine and psychometric evaluation of a placebo version of the “Trier Social Stress Test”. Psychoneuroendocrinology. (2009) 34:1075–86. doi: 10.1016/j.psyneuen.2009.02.008, PMID: 19307062

[87] Simonovic B Stupple EJN Gale M Sheffield D. Stress and risky decision making: cognitive reflection, emotional learning or both. J Behav Decis Mak. (2017) 30:658–65. doi: 10.1002/bdm.1980

[88] Simonovic B Stupple EJN Gale M Sheffield D. Performance under stress: an eye-tracking investigation of the Iowa Gambling Task (IGT). Front Behav Neurosci. (2018) 12:217. doi: 10.3389/fnbeh.2018.00217, PMID: 30319368 PMC6166123

[89] Wemm SE Wulfert E. Effects of acute stress on decision making. Appl Psychophysiol Biofeedback. (2017) 42:1–12. doi: 10.1007/s10484-016-9347-8, PMID: 28083720 PMC5346059

[90] Miu AC Heilman RM Houser D. Anxiety impairs decision-making: Psychophysiological evidence from an Iowa Gambling Task. Biol Psychol. (2008) 77:353–8. doi: 10.1016/j.biopsycho.2007.11.010, PMID: 18191013

[91] Ben Hassen N Molins F Paz M Serrano M-Á. Later stages of acute stress impair reinforcement-learning and feedback sensitivity in decision making. Biol Psychol. (2023) 180:108585. doi: 10.1016/j.biopsycho.2023.108585, PMID: 37178755

[92] Singh V. Role of cortisol and testosterone in risky decision-making: deciphering male decision-making in the Iowa Gambling Task. Front Neurosci. (2021) 15:631195/BIBTEX. doi: 10.3389/FNINS.2021.631195/BIBTEX, PMID: 34211361 PMC8239136

[93] Cauffman E Shulman EP Steinberg L Claus E Banich MT Graham S. Age differences in affective decision making as indexed by performance on the Iowa Gambling Task. Dev Psychol. (2010) 46:193–207. doi: 10.1037/a0016128, PMID: 20053017

[94] Smith DG Xiao L Bechara A. Decision making in children and adolescents: impaired Iowa Gambling Task performance in early adolescence. Dev Psychol. (2012) 48:1180–7. doi: 10.1037/a0026342, PMID: 22081879

[95] Crone EA van der Molen MW. Developmental changes in real life decision making: performance on a gambling task previously shown to depend on the ventromedial prefrontal cortex. Dev Neuropsychol. (2004) 25:251–79. doi: 10.1207/s15326942dn2503_2, PMID: 15147999

[96] Toplak ME Sorge GB Benoit A West RF Stanovich KE. Decision-making and cognitive abilities: A review of associations between Iowa Gambling Task performance, executive functions, and intelligence. Clin Psychol Rev. (2010) 30:562–81. doi: 10.1016/j.cpr.2010.04.002, PMID: 20457481

[97] Vermaercke B Cop E Willems S D’Hooge R Op de Beeck HP. More complex brains are not always better: rats outperform humans in implicit category-based generalization by implementing a similarity-based strategy. Psychon Bull Rev. (2014) 21:1080–6. doi: 10.3758/s13423-013-0579-9, PMID: 24408657

[98] Hultman C Tjernström N Vadlin S Rehn M Nilsson KW Roman E. Exploring decision-making strategies in the Iowa gambling task and rat gambling task. Front Behav Neurosci. (2022) 16:964348. doi: 10.3389/fnbeh.2022.964348, PMID: 36408452 PMC9669572

[99] Arnsten AFT. Stress weakens prefrontal networks: molecular insults to higher cognition. Nat Neurosci. (2015) 18:1376–85. doi: 10.1038/nn.4087, PMID: 26404712 PMC4816215

[100] Kalueff AV Murphy DL. The importance of cognitive phenotypes in experimental modeling of animal anxiety and depression. Neural Plast. (2007) 2007:52087. doi: 10.1155/2007/52087, PMID: 18288249 PMC2233771

[101] Deslauriers J Toth M Der-Avakian A Risbrough VB. Current status of animal models of posttraumatic stress disorder: behavioral and biological phenotypes, and future challenges in improving translation. Biol Psychiatry. (2018) 83:895–907. doi: 10.1016/j.biopsych.2017.11.019, PMID: 29338843 PMC6085893

[102] Catalán-Aguilar J González-Bono E Lozano-García A Tormos-Pons P Hampel KG Villanueva V. Stress phenotypes in epilepsy: impact on cognitive functioning and quality of life. Front Psychol. (2023) 14:1100101. doi: 10.3389/fpsyg.2023.1100101, PMID: 37388654 PMC10300421

[103] Starcke K Brand M. Decision making under stress: a selective review. Neurosci Biobehav Rev. (2012) 36:1228–48. doi: 10.1016/j.neubiorev.2012.02.003, PMID: 22342781

[104] Bolla KI Eldreth DA Matochik JA Cadet JL. Sex-related differences in a gambling task and its neurological correlates. Cereb Cortex. (2004) 14:1226–32. doi: 10.1093/CERCOR/BHH083, PMID: 15142963

[105] Nicoletti A Baschi R Cicero CE Iacono S Re VL Luca A. Sex and gender differences in Alzheimer’s disease, Parkinson’s disease, and Amyotrophic Lateral Sclerosis: A narrative review. Mech Ageing Dev. (2023) 212:111821. doi: 10.1016/j.mad.2023.111821, PMID: 37127082

[106] Beitz KM Salthouse TA Davis HP. Performance on the Iowa Gambling Task: from 5 to 89 years of age. J Exp Psychol Gen. (2014) 143:1677–89. doi: 10.1037/a0035823, PMID: 24512562 PMC4115037

[107] Holmes PV. Rodent models of depression: reexamining validity without anthropomorphic inference. Crit Rev Neurobiol. (2003) 15:143–74. doi: 10.1615/critrevneurobiol.v15.i2.30, PMID: 14977368

[108] van der Staay FJ Arndt SS Nordquist RE. Evaluation of animal models of neurobehavioral disorders. Behav Brain Funct. (2009) 5:11. doi: 10.1186/1744-9081-5-11, PMID: 19243583 PMC2669803

[109] Nestler EJ Hyman SE. Animal models of neuropsychiatric disorders . Nat Neurosci. (2010) 13:1161–9. doi: 10.1038/nn.2647, PMID: 20877280 PMC3750731

[110] Clayton JA Collins FS. Policy: NIH to balance sex in cell and animal studies. Nature. (2014) 509:282–3. doi: 10.1038/509282a, PMID: 24834516 PMC5101948

[111] Voskuhl R Klein S. Sex is a biological variable — in the brain too. Nature. (2019) 568:171–1. doi: 10.1038/d41586-019-01141-6, PMID: 30967673

[112] Cross CP Copping LT Campbell A. Sex differences in impulsivity: a meta-analysis. psychol Bull. (2011) 137:97. doi: 10.1037/a0021591, PMID: 21219058

[113] Singh V Grewal KS Vibha D Singh RK Ramanujam B Nehra A. Cortico-limbic disruption, material-specificity, and deficits in cognitive-affective theory of mind. Brain Commun. (2023) 5(2):fcad100. doi: 10.1093/braincomms/fcad100, PMID: 37101833 PMC10123397

